# MclX modulates the process of mother cell lysis by SpoIIID in *Bacillus thuringiensis*

**DOI:** 10.1128/aem.00215-26

**Published:** 2026-08-11

**Authors:** Yanrong Xu, Xin Zhang, Ruibin Zhang, Dongshu Wang, Shaomi Dai, Xiaowen Du, Shuyuan Guo, Fuping Song

**Affiliations:** 1 State Key Laboratory for Biology of Plant Diseases and Insect Pests, Institute of Plant Protection, Chinese Academy of Agricultural Sciences 243827, Beijing, China; 2 College of Life Science, Northeast Agricultural University 12430, Harbin, China; 3 College of Life Sciences, Hubei University 12563, Wuhan, China; 4 State Key Laboratory of Pathogens and Biosecurity, Academy of Military Medical Sciences 71040, Beijing, China; 5 School of Life Science, Beijing Institute of Technology 428678, Beijing, China; Michigan State University, East Lansing, Michigan, USA

**Keywords:** cell differentiation, mother cell lysis process, transcriptional regulator SpoIIID, cell wall hydrolase gene *cwlC*, unknown gene *mclX*

## Abstract

Cell differentiation is essential for the survival and adaptation of organisms. In *Bacillus* species, sporulation is a tightly coordinated program that culminates in mother cell lysis, a critical step for spore release. Despite its importance, the mechanisms modulating the process remain poorly understood. Here, we show that MclX regulates mother cell lysis by inhibiting SpoIIID, a sporulation-specific transcription factor that represses *cwlC*, which encodes a key hydrolase required for lysis. SpoIIID directly binds to the *cwlC* promoter, and its overexpression during sporulation markedly delays both the onset and progression of mother cell lysis. These findings support a model in which MclX modulates SpoIIID to control *cwlC* expression and lysis process. Collectively, this work defines a previously unrecognized MclX–SpoIIID–*cwlC* regulatory axis, providing new insight into the control of late sporulation events and the process of mother cell lysis.

**IMPORTANCE:**

Mother cell lysis is a critical step in *Bacillus* sporulation, yet its regulation remains poorly understood. Here, we identify *mclX* as an upstream factor that modulates the onset and progression of mother cell lysis in *Bacillus thuringiensis*. MclX reduces SpoIIID levels, which in turn directly represses *cwlC* expression. These findings provide new insight into the regulatory network governing lysis process and advance our understanding of bacterial cell differentiation.

## INTRODUCTION

Cell development is a fundamental strategy for microbial survival and adaptation, involving diverse processes, such as differentiation, growth, and programmed cell death (1–3). Sporulation is a fundamental developmental program in *Bacillus* species, including asymmetric separation, engulfment, cortex formation, coat formation, and mother cell lysis (4, 5). A critical feature of this process is mother cell lysis, which enables the release of mature spores into the environment (6, 7). The spore ensures long-term survival under harsh conditions and provides a widely studied model for bacterial cell differentiation (8, 9). This process involves a cascade of morphological transitions and a hierarchically regulated transcriptional network, making it one of the best-studied models of prokaryotic development (4, 10, 11). Despite decades of research, several key steps remain incompletely understood, particularly the mechanism of mother cell lysis, the terminal event of sporulation that ensures spore release. While multiple transcription factors and cell wall hydrolases have been implicated, the regulatory coordination and precise timing of lysis remain as open questions (12, 13).

Mother cell lysis is controlled by peptidoglycan hydrolases under strict transcriptional regulation. CwlB, CwlC, and CwlH function redundantly in *Bacillus subtilis* (*B. subtilis*), as single deletions allow lysis; double deletions block it; and triple deletions only delay the process (14–16). By contrast, in *Bacillus thuringiensis* (*B. thuringiensis*), deletion of *cwlB* delays mother cell lysis, whereas deletion of *cwlC* completely abolishes this process (17, 18). The expression of the genes encoding these hydrolases is governed by σ^K^ and modulated by GerE, linking their activity to late-stage mother cell development (14–19). In *B. subtilis*, these expression differences are controlled by a transcriptional cascade. σ^E^ activates the expression of the *spoIIID* gene, and the resulting SpoIIID transcription factor transcripts and activates the expression of the gene encoding σ^K^ (10, 20). SpoIIID is a small DNA-binding protein that regulates more than 100 genes, acting as both activator and repressor in σ^E^ and σ^K^ regulons (21). Although this network is well defined, the regulatory pathway that controls mother cell lysis remains unknown.

*B. thuringiensis* represents a valuable model system to address these knowledge gaps. *B. subtilis* is widely used to study sporulation and is largely nonpathogenic. In contrast, *B. thuringiensis* couples spore formation with the production of insecticidal crystal proteins. This makes the regulation of mother cell lysis and subsequent spore directly relevant to both ecological adaptation and its use as a bioinsecticide. In *B. thuringiensis*, SpoIIID is known to influence mother cell lysis and positively regulates *sigK* transcription (22). However, its specific contribution to mother cell lysis remains unclear. Moreover, deletion of *mclX*, a previously uncharacterized gene, blocks mother cell lysis, and MclX indirectly activates transcription of the key hydrolase gene *cwlC* (17, 23). Notably, its homolog YodN has been characterized in *B. subtilis* and implicated in spore germination (24). Although both MclX and *cwlC* are required for mother cell lysis, the regulatory mechanism linking MclX to *cwlC* has not been elucidated.

In this study, we determined that *mclX* is expressed earlier than *cwlC* and that MclX negatively regulates SpoIIID levels. We further showed that SpoIIID directly represses *cwlC* transcription and that heterologous overexpression of *spoIIID* significantly delays mother cell lysis. Thus, these findings demonstrate, for the first time, that MclX modulates the process of mother cell lysis through SpoIIID, providing mechanistic and practical insights into *B. thuringiensis* cell differentiation and biopesticide optimization.

## MATERIALS AND METHODS

### Bacterial strains, plasmids, and growth conditions

All bacterial strains and plasmids used in this study are listed in Table 1. *Escherichia coli* strains TGI and BL21 (DE3) were used as hosts for molecular cloning and protein expression, respectively. *E. coli* SCS110 (also called ET12567) (25) was used to generate unmethylated plasmid DNA for transformation into *B. thuringiensis* cells (26). All *E. coli* strains were cultured at 37°C in Luria-Bertani (LB) medium (1% NaCl, 1% tryptone, and 0.5% yeast extract) or on LB agar plates (LB medium supplemented with 1.3% agar). Ampicillin (100 µg/mL) was added when necessary. The wild-type *B. thuringiensis* subsp. *kurstaki* strain HD73 (GenBank Accession Number: NC_020238.1) was used as the recipient strain for gene expression analysis and genetic manipulation (27, 28). HD73 and its derivatives were routinely cultured at 30°C in LB medium or on LB agar plates supplemented with erythromycin (5 µg/mL) or kanamycin (100 µg/mL) when required. *B. thuringiensis* strains were cultured in Schaeffer’s sporulation medium (SSM; 0.8% nutrient broth, 0.1% KCl, 0.025% MgSO_4_·7H_2_O, 0.0002% MnCl_2_·4H_2_O, 0.5 mM CaCl_2_, 0.001 mM FeSO_4_; pH 7.0) (29) with continuous shaking (220 rpm) at 30°C.

**TABLE 1:** Functional classification of transcriptional regulators among upregulated proteins in Δ*mclX* at T12

Gene ID	Annotation	SD^*a*^
HD73_RS28325	Sporulation transcriptional regulator SpoIIID	3.93
HD73_RS23130	RNA polymerase sporulation sigma factor SigK	2.5
HD73_RS20925	RsfA family transcriptional regulator	2.13
HD73_RS22890	RNA polymerase sigma factor RpoD	1.26

^*a*^ SD (Student’s *t*-test difference) represents the fold change in protein expression of the mutant strain Δ*mclX* relative to the wild-type HD73 in the proteomic analysis.

### DNA manipulation and transformation

Plasmid DNA was extracted from *E. coli* cells (listed in Table 2) using a Plasmid Mini Kit I (Omega Bio-Tek, Guangzhou, China). PCR amplification was carried out with Taq DNA polymerase (Vazyme, Nanjing, China) or PrimeSTAR HS DNA polymerase (TaKaRa Biotechnology, Beijing, China). DNA fragments were purified using the Gel Extraction Kit (Omega Bio-Tek, USA). Genomic DNA from *B. thuringiensis* HD73 served as the template for all PCR reactions. Oligonucleotide primers (Table 2) were synthesized by Sangon (Shanghai, China). Restriction enzymes and T4 DNA ligase (TaKaRa, Dalian, China) were used according to the manufacturer’s instructions. All recombinant constructs were verified by DNA sequencing (BGI, Beijing, China). Standard transformation procedures for *E. coli* (30) and *B. thuringiensis* were performed as described previously.

**TABLE 2:** Strains and plasmids used in this study

Strain or plasmid	Relevant details^*a*^	Source or reference
Strains		
*E. coli* TG1	Δ(*lac*-*proAB*) *supE thi hsd-5* (*F’ traD36 proA* + *proB* + *lacIq lacZ*ΔM15), general purpose cloning host	Laboratory
*E. coli* ET 12567	*F*^*-*^ *dam-13*::Tn nine *dcm*-*6 hsdM hsdR recF143 zjj*-*202*::Tn10 *galK2 galT22 ara14 pacY1 xyl*-*5 leuB6 thi*-*1*, for generation of unmethylated DNA	Laboratory
BL21(DE3)	*F*^-^ dcmopmT hsds (r_B_^-^ m_B_^-^) galλ(DE3)	(31)
BL21(pET*spoIIID*)	BL21(DE3) with pET*spoIIID* plasmid	This study
HD73	*Bacillus thuringiensis* serovar *kurstaki* wild-type strain containing plasmid pHT73 carrying *cry1Ac* gene	Laboratory
∆*mclX*	HD73 mutant, *mclX* gene was deleted by homologous recombination	(23)
∆*cwlC*	HD73 mutant, *cwlC* gene was deleted by homologous recombination	(17)
∆*spoIIID*	HD73 mutant, *spoIIID* gene was deleted by homologous recombination	(32)
∆*spoIIID*∆*cwlC*	HD73 mutant, *spoIIID* and *cwlC* genes were deleted by homologous recombination	
∆*spoIIID*∆*mclX*	HD73 mutant, *spoIIID* and *mclX* genes were deleted by homologous recombination	This study
HD(P*mclX*-*lacZ*)	HD73 strain containing plasmid pHT304-P*mclX*-*lacZ*	(23)
HD(P*cwlC*-*lacZ*)	HD73 strain containing plasmid pHT304-P*cwlC*-*lacZ*	(17)
HD(P*spoIIID*-*lacZ*)	HD73 strain containing plasmid pHT304-P*spoIIID*-*lacZ*	This study
HD(P*exsB*-*lacZ*)	HD73 strain containing plasmid pHT304-P*exsB*-*lacZ*	(33)
HD(P*exsY*-*lacZ*)	HD73 strain containing plasmid pHT304-P*exsY*-*lacZ*	(34)
HD(P*spoIIID*)	HD73 strain containing plasmid pHT1618-P*spoIIID*-*spoIIID*	This study
HD(P*exsB*)	HD73 strain containing plasmid pHT1618-P*exsB*-*spoIIID*	This study
HD(P*exsY*)	HD73 strain containing plasmid pHT1618-P*exsY*-*spoIIID*	This study
*B. subtilis* 168	Expression strain; *Bacillus subtilis*	Laboratory
Bs∆*yodN*	*B. subtilis* 168 mutant, *yodN* gene was deleted	This study
Plasmids		
pHT304-P*exsB*-*lacZ*	pHT304-18Z carrying P*exsB*, Amp^R^, Erm^R^	(33)
pHT304-P*exsY*-*lacZ*	pHT304-18Z carrying P*exsY*, Amp^R^, Erm^R^	(34)
pHT304-P*spoIIID*-*lacZ*	pHT304-18Z carrying P*spoIIID*, Amp^R^, Erm^R^	This study
pHT1618-P*spoIIID*-*spoIIID*	pHT1618 carrying P*spoIIID* and *spoIIID* gene	This study
pHT1618-P*exsB*-*spoIIID*	pHT1618 carrying P*exsB* and *spoIIID* gene	This study
pHT1618-P*exsY*-*spoIIID*	pHT1618 carrying P*exsY* and *spoIIID* gene	This study
pJOE8999	pJOE8999 carrying P*manP*, Cas9, PvanP*, gRNA, Kan^R^	(35)
pJOE8999-sgRNA-*yodN*	pJOE8999 carrying P*manP*, Cas9, PvanP*, gRNA-*yodN*, Kan^R^	This study

^*a*^ Antibiotic resistance cassettes are indicated as follows: Erm^R^, erythromycin resistance; Kan^R^, kanamycin resistance; Amp^R^, ampicillin resistance.

### Construction of the *mclX* and *spoIIID* deletion mutant

To construct the *spoIIID* and *mclX* double-deletion mutant strain, a homologous recombination approach was employed. The recombinant plasmid pMAD-*mclX* (23) was transformed into the Δ*spoIIID* competent cells by electroporation. Transformants were selected on LB agar plates containing kanamycin. Colonies that grew on kanamycin-resistant LB agar plates but failed to grow on erythromycin-resistant LB agar plates were screened. The Δ*spoIIID*Δ*mclX* double mutant was verified by PCR and sequencing with the primers *mclX*-F/*mclX*-R.

### Construction of *spoIIID*-overexpression strains

To construct *spoIIID*-overexpression strains, a series of promoter–gene fusion fragments were generated by PCR using *B. thuringiensis* HD73 genomic DNA as the template. Promoters were selected based on their reported activity during sporulation: P*spoIIID* approximates native expression; P*exsB* is active at late stages; and P*exsY* exhibits sustained high transcriptional activity during late sporulation (33, 34). All overexpression constructs were generated using the plasmid pHT1618 and introduced into *B. thuringiensis* by electroporation. A 808 bp fragment containing the *spoIIID* open reading frame (ORF) and its native promoter P*spoIIID* was amplified using primers P*spoIIID-spoIIID*-F/P*spoIIID-spoIIID*-R. For the P*exsB-spoIIID* construct, two overlapping fragments were amplified. The P*exsB* promoter fragment (577 bp) was amplified with the P*exsB*-F/P*exsB*-R primers, and the *spoIIID* fragment (318 bp) was amplified with the P*exsB-spoIIID*-F/P*exsB-spoIIID*-R primers. The above two fragments were fused by overlapping PCR with primers P*exsB*-F/P*exsB-spoIIID*-R to generate an 840 bp P*exsB-spoIIID* fragment. Similarly, for the P*exsY-spoIIID* construct, the P*exsY* promoter fragment (603 bp) was amplified with the P*exsY*-F/P*exsY*-R primers, and the *spoIIID* fragment (317 bp) was amplified with the P*exsY-spoIIID*-F/P*exsY-spoIIID*-R primers. The two fragments were combined by overlapping PCR using primers P*exsY*-F/P*exsY-spoIIID*-R to obtain an 866 bp P*exsY-spoIIID* fragment. The 808 (P*spoIIID-spoIIID*), 840 (P*exsB-spoIIID*), and 866 bp (P*exsY-spoIIID*) fragments were individually digested with HindIII and PstI, ligated into the similarly digested pHT1618 vector (36). The recombinant plasmids pHT1618-P*spoIIID-spoIIID*, pHT1618-P*exsB-spoIIID*, and pHT1618-P*exsY-spoIIID* were introduced into *B. thuringiensis* HD73 by electroporation to generate the strains HD(P*spoIIID*), HD(P*exsB*), and HD(P*exsY*), respectively, for *spoIIID* overexpression.

### Construction of promoters with *lacZ* gene fusions

To compare the transcriptional activities of different promoters in the *B. thuringiensis* HD73 wild-type and related strains, the promoter region of *spoIIID* (P*spoIIID*, 535 bp) was amplified by PCR using *B. thuringiensis* genomic DNA as the template. All primers used are listed in Table 3. The amplified fragments were digested with PstI and BamHI, purified by gel extraction, and ligated into the similarly digested pHT304-18Z vector (37), which contains a promoter-less *lacZ* gene. The resulting recombinant plasmids P*spoIIID-lacZ* were introduced into *B. thuringiensis* HD73 and the Δ*mclX* mutant to generate HD(P*spoIIID-lacZ*) and Δ*mclX*(P*spoIIID-lacZ*) strains, respectively. Similarly, the plasmid P*cwlC-lacZ* (17) was transformed into HD73 and the *spoIIID*-overexpression strain HD(P*exsY-spoIIID*), respectively, producing HD(P*cwlC-lacZ*) and HD(P*exsY-spoIIID*) (P*cwlC-lacZ*) strains. In addition, the plasmids P*mclX-lacZ* (23), P*exsB-lacZ* (33), and P*exsY-lacZ* (34) were individually introduced into HD73, obtaining the strains HD(P*mclX-lacZ*), HD(P*exsB-lacZ*), and HD(P*exsY-lacZ*).

**TABLE 3:** Oligonucleotide primers used in this study

Primer name	Sequence (5′−3′)	Restriction site
*mclX*-1	AGATCTATCGATGCATGCCATGGTACCCGGGAGGACACCATGTATTTCTTGTTCCTCG	
*mclX*-6	GTCTGCAGAAGCTTCTAGAATTCGAGCTCCGCCCTCTACGACGATAATCACTAC	
*mclX*-F	GAGAGCTATATTCCAGGAAGAGCGG	
*mclX*-R	CGACATATGTAACCCCATAACTGCCC	
pMAD-F	GCCATTGCTCTGGGTTATA	
pMAD-R	TCATCTACCTGCCTGGACA	
*cwlC*-F	CGGGATCCGACTACGGACCGATGGAAGC	
*cwlC*-R	GTTTGAACCATCTTGCACACGT	
PU	CGCCAGGGTTTTCCCAGTCAC	
PR	AGCGGATAACAATTTCACACAGGA	
OVG	GTAATCTTACGTCAGTAACTTCCACAGTA	
P*spoIIID*-F	AACTGCAGATATAAAAATACTCAATTTCGAG	PstI
P*spoIIID*-R	CGGGATCCACCACTCGCCTCCCTAATTTGG	BamHI
P*spoIIID-spoIIID*-F	CCCAAGCTTATATAAAAATACTCAATTTCGAGAAAAAAGTTGCAATAAC	HindIII
P*spoIIID-spoIIID*-R	AACTGCAGTTATTGACGTACAGGCTTCTCTACATC	PstI
P*exsB*-F	CCCAAGCTTTGAAACTCTTTCGCACCTTGAAATTTT	HindIII
P*exsB*-R	AATCGTGCACACCACTCGCCTCCCTTTATATTCTTGGGCATTTACAATCTTCGTTACA	
P*exsB-spoIIID*-F	AACGAAGATTGTAAATGCCCAAGAATATAAAGGGAGGCGAGTGGTG	
P*exsB-spoIIID*-R	AACTGCAGTTATTGACGTACAGGCTTCTCTACATC	PstI
P*exsY*-F	CCCAAGCTTCGGTTCCGCAACGATAGG	HindIII
P*exsY*-R	CTGCATCAGTAGCAAATACACGCCCATAAAGGGAGGCGAGTGGTGTGCACGATT	
P*exsY-spoIIID*-F	CTGCATCAGTAGCAAATACACGCCCATAAAGGGAGGCGAGTGGTGTGCACGATTA	
P*exsY-spoIIID*-R	AACTGCAGTTATTGACGTACAGGCTTCTCTACATCT	PstI
pET*spoIIID*-F	CGGATCCATGCACGATTACATCAAAGAGAGAACT	BamHI
pET*spoIIID*-R	ACGCGTCGACTTGACGTACAGGCTTCTCTACATCT	SalI
*spoIIID*-F	CACAATCGGAAGATCCAGCAG	
*spoIIID*-R	GTCGATGATATCTGCAGATAATTC	
*cry1Ac*-F	ATGGATAACAATCCGAACATCAATGAAT	
*cry1Ac*-R	TCCATAAGCAAATTCTGTCCCGTCAAGA	
P*cwlC*-F-EMSA	CATGCGGAAAAAAGAAAATTT	
P*cwlC*-R-FAM	TTGTGTCCTCCGTGCAAACT	
RT*spoIIID*-F	AAGACAGTGCGTGTCATTGC	
RT*spoIIID*-R	CACTTCATTTGCGAGCTCTGG	
RT*16S*-F	TCGCATTAGCTAGTTGGTGAG	
RT*16S*-R	TCTTCCCTAACAACAGAGTTT	
pJOE8999-*yodN*-N20-F	CCCGTTTAAAGGATGGAGAGGTTTTAGAGCTAGAAATAGCAAGTTAAAATAAGG	
pJOE8999-*yodN*-N20-R	CTCTCCATCCTTTAAACGGGCGTAGGTACATTTTACTCAATTCTCTAATC	
*yodN*-5HA-F	CACTCATCTTCATCCAGCTCT	
*yodN*-5HA-R	GTGTCAATTGTTGATGAGCTTCT	
*yodN*-3HA-F	AGAAGCTCATCAACAATTGACACCTCGCCGCAACATTTCTCATA	
*yodN*-3HA-R	GCCTGAAAGCCTGACATATCA	
*yodN*-F	TATGAATGGCCGCAGCTAG	
*yodN*-R	CCTGCCATGGTATGTTCTTC	

### Western blot analysis

Western blotting was performed as previously described (38). Briefly, total protein samples were separated by SDS-PAGE using a 4% polyacrylamide stacking gel and a 15% separating gel. The separated proteins were then transferred onto a polyvinylidene difluoride (PVDF) membrane. The membranes were blocked and subsequently incubated with a primary antibody against SpoIIID (anti-SpoIIID, rabbit polyclonal antibody; Wuhan GeneCreate Biological Engineering Co., Ltd., China). Antibody binding was detected using an HRP-conjugated goat anti-rabbit IgG secondary antibody (EASYBIO, Beijing, China).

### Electrophoresis mobility shift assays

DNA fragments containing the P*cwlC* promoter region were amplified from *B. thuringiensis* HD73 genomic DNA using specific primers (P*cwlC*-F-EMSA/P*cwlC*-R-FAM), with a 6-FAM fluorescent label at the 5′ end. Electrophoretic mobility shift assays (EMSA) were performed as previously described (39) to examine the interaction between purified SpoIIID protein and the P*cwlC* promoter fragments.

### β-Galactosidase activity assays

The experiments were performed in SSM medium (40). The end of the exponential phase was designated as T0, and Tn represented *n* hours after T0. Samples (2 mL) were collected at 1 h intervals. They were centrifuged at 12,000 ×*g* for 1 min, and the supernatants were removed. β-Galactosidase activity was determined as previously described (5) and expressed in Miller units. Reported values represent the mean ± standard error from at least three independent experiments.

### Expression and purification of SpoIIID-His protein

The *spoIIID* gene from *B. thuringiensis* HD73 was amplified using primers pET*spoIIID*-F and pET*spoIIID*-R, and the PCR product was cloned into the BamHI and SalI sites of the pET21b vector to generate pET*spoIIID*. The plasmid was transformed into *E. coli* BL21(DE3). Transformants were cultured in LB medium containing ampicillin (100 μg/mL) at 37°C until the exponential phase. SpoIIID-His protein expression was induced with 1 mM isopropyl β-D-1-thiogalactopyranoside (IPTG) for 12 h at 18°C. The recombinant SpoIIID-His protein was purified according to previously described procedures (40).

### Microscope observation

Cells were grown in the appropriate medium, and samples were collected at the indicated time points. Transmission electron microscopy (TEM) was performed using a HITACHI HT7800 electron microscope as previously described (38). Optical microscopy was conducted with an Olympus BX63 microscope in bright-field mode. Data were obtained from at least three independent experiments.

### Determination of cell number

Samples of *B. thuringiensis*, including the HD73 wild-type and *spoIIID*-overexpression strains HD(P*spoIIID-spoIIID*), HD(P*exsB-spoIIID*), and HD(P*exsY-spoIIID*), were cultured in SSM medium. The numbers of lysed and non-lysed cells were counted from optical microscopy images. At least 500 cells were quantified for each strain. The mother cell lysis rate was defined as the ratio of the number of lysed cells to the total number of cells. Data were obtained from three independent experiments.

### Sporulation efficiency

Sporulation efficiency was measured in SSM medium. *B. thuringiensis* cells cultured for T24 were collected. Heated (65°C for 30 min) and unheated samples were serially diluted and plated onto LB agar plates. Sporulation frequency was calculated as the ratio of heat-resistant colonies to total colonies. Error bars represent three independent experiments.

### Mass spectrometry sample preparation and analysis

The experiments were performed in SSM medium at 30°C. Samples of HD73 and ∆*mclX* were harvested at T12 to analyze the differentially expressed proteins. Protein concentrations were measured using a 2-D Quant Kit (GE Healthcare, USA). The extracted protein samples were enriched in ultrafiltration tubes (0.5 mL, 10 kD; Thermo), washed three times with 8 M urea, and alkylated with 20 mM iodoacetamide in the dark for 30 min at room temperature. Columns were then washed with 8 M urea, followed by 50 mM NH_4_HCO_3_ three times to remove the urea. Trypsin was added to the columns, which were then incubated at 37°C for 13 h. The digested peptides were harvested into fresh tubes, dried via vacuum concentration after mixing with 100% formic acid, and then dissolved again in 0.1% formic acid. The samples were further analyzed by liquid chromatography-tandem mass spectrometry (LC-MS/MS). The nano LC system was used for peptide separation, and eluted peptides were analyzed using an Orbitrap Lumos fusion mass spectrometer (Thermo Fisher Scientific) in accordance with the manufacturer’s instructions.

All spectra were searched against the *B. thuringiensis* HD73 genome (NC_020238.1) obtained from GenBank using the PD 2.1 (Proteome Discoverer, Thermo Fisher Scientific) computational platform. The whole experiments were repeated three times. The differentially expressed proteins were subjected to the Kyoto Encyclopedia of Genes and Genomes (KEGG) pathway enrichment analysis using an online gene function analysis tool.

### Real-time quantitative PCR assay

The experiments were performed in SSM medium. Samples of HD73 and ∆*mclX* were harvested at T8, T12, T16, and T20, with three independent biological replicates, to analyze the expression levels of *spoIIID*. Total RNA was extracted using the RNAprep Pure Kit (Aidlab, Beijing, China). cDNA was synthesized by reverse transcription with the HiScript II Q RT SuperMix (Vazyme, Nanjing, China). The transcription levels of *spoIIID* were measured by real-time quantitative PCR (RT-qPCR) using the ChamQ Universal SYBR qPCR Master Mix (Vazyme). The 16S rRNA gene served as an internal control. Primer sequences used for RT-qPCR are listed in Table 2.

### Construction of the *yodN* gene knockout mutant of *B. subtilis* 168

To delete the *yodN* gene in the *B. subtilis* 168 genome, the CRISPR/Cas9-based plasmid pJOE8999 was used as the editing vector (the plasmids and strains used in this study are listed in Table 1). The plasmid pJOE8999-sgRNA-*yodN* was constructed, which contained the promoters P*manP* and P*vanP*, the Cas9 gene, the gRNA-*yodN* expression cassette, and a kanamycin resistance gene for selection. To introduce the *yodN*-targeting guide sequence (N20) into the plasmid, the primers pJOE8999-*yodN*-N20-F and pJOE8999-*yodN*-N20-R were designed (all primers used in this study are listed in Table 3). The homologous arms flanking *yodN* were amplified from *B. subtilis* 168 genomic DNA using the primer pairs *yodN*-5HA-F/R and *yodN*-3HA-F/R. The two fragments were then fused by overlapping extension PCR to generate a 5′−3′ homologous arm (HA) repair template. During transformation, the recombinant plasmid pJOE8999-sgRNA-*yodN* and the fused HA fragment were co-transformed into competent *B. subtilis* 168 cells. The plasmid mediated Cas9-induced double-strand cleavage at the *yodN* locus, while the HA fragment served as the donor DNA for homologous recombination repair (41). Transformation was performed at 37°C and 220 rpm for 1.5 h using a thermostatic shaker (ZQZY-CF, Zhichu, Shanghai, China) and an incubator (GHP-9080, Yiheng, Shanghai, China). The cells were then spread on LB agar plates containing kanamycin (20 μg/mL) and 0.2% mannose and incubated overnight at 30°C. Transformants were selected, and the resulting colonies were verified by PCR using primers *yodN*-F and *yodN*-R. The deletion mutant BsΔ*yodN* was confirmed to have a 563 bp deletion (from nucleotide 2,138,166 to 2,138,728 on the *B. subtilis* 168 genome).

### Statistical analysis

Statistical analyses were conducted using Microsoft Excel and GraphPad Prism version 9.1. Data are presented as mean values with the standard error of the mean. Differences between groups were assessed using the Student’s *t*-test and considered significant at *P*  <  0.05. Levels of significance are indicated as follows: * for *P*  <  0.05, ** for *P*  <  0.01, *** for *P*  <  0.001, and **** for *P*  <  0.0001. All experiments included at least three biological replicates.

## RESULTS

### Deletion of *mclX* results in upregulation of SpoIIID expression

**Fig 1:**
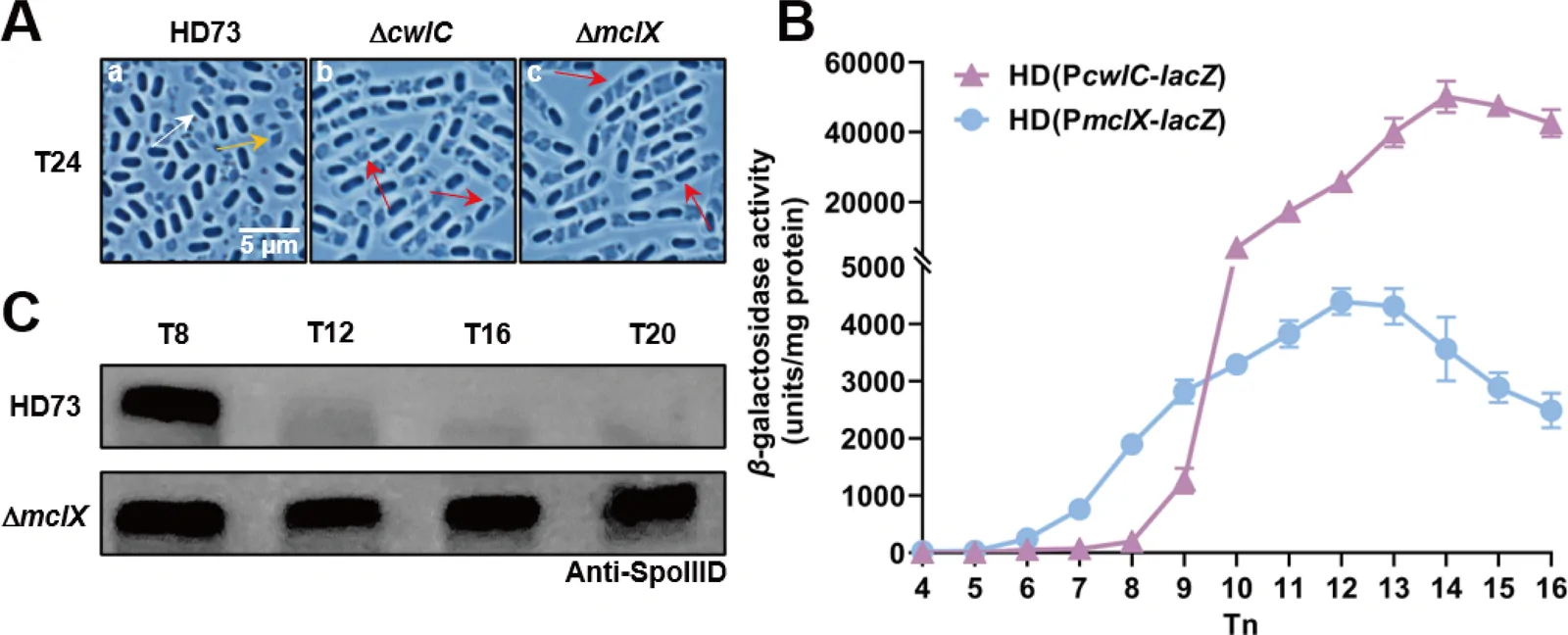
*mclX* deletion upregulates SpoIIID expression. (**A**) Micrographs of the HD73 wild-type, Δ*cwlC*, and Δ*mclX* strains. Δ*mclX* represents the *mclX* deletion mutant strain, and Δ*cwlC* represents the *cwlC* deletion mutant strain. All strains were grown at 30°C in SSM medium and imaged using an Olympus BX63 microscope in bright-field mode. Three structures are indicated: spores (white arrows), parasporal crystals (yellow arrows), and intact mother cells containing spores and crystals (red arrows). Lysed cells are identified by the release and dispersion of spores and crystals into the surrounding field, whereas in non-lysed cells, these structures remain enclosed within the mother cell. Scale bar, 5 μm. (**B**) Transcription analysis of the *cwlC* (P*cwlC*) and *mclX* (P*mclX*) promoters in wild-type HD73. The β-galactosidase activities of three clones were determined at specified time points after cultivating *B. thuringiensis* cells in SSM at 30°C. Each value represents the mean and standard error of at least three independent replicates. (**C**) Western blot analysis of SpoIIID expression dynamics in HD73 and Δ*mclX*. All strains were grown at 30°C in SSM with shaking. Cells were harvested at T8, T12, T16, and T20. SpoIIID antibody (Anti-SpoIIID) was used to detect the expression level of SpoIIID.

Previous studies have demonstrated that deletion of either *cwlC* or *mclX* prevented mother cell lysis, whereas the wild-type strain HD73 lysed after T24 (Fig. 1A). The transcription of *cwlC* and *mclX* was examined by fusing their respective promoters (P*cwlC* and P*mclX*) to the *lacZ* reporter gene and measuring β-galactosidase activity in the background of the wild-type strain HD73 . The β-galactosidase assays revealed that P*mclX* began transcription at T6 and peaked at T12, while P*cwlC* began transcription at T8 and reached maximum activity at T14 (Fig. 1B). Thus, if CwlC and MclX are functionally related, then *mclX* may function upstream of *cwlC*.

To investigate how MclX indirectly affects *cwlC* transcription (17, 23), we performed proteomic analysis of HD73 and Δ*mclX* at T12, when *mclX* transcription peaks and precedes maximal *cwlC* expression (T14) (Fig. 1B), representing a key regulatory stage. Differentially expressed proteins were identified based on a Student’s *t*-test difference >1 and a false discovery rate cutoff of *P* < 0.05 using proteomic data. The analysis revealed 188 proteins with differential expression in Δ*mclX* relative to HD73. Among these proteins, 116 were upregulated, and 72 were downregulated. To explore the functional roles of these differentially expressed proteins, they were classified related to amino acid metabolism, signal transduction, cellular processing, sporulation, transcriptional regulators, and mother cell lysis according to KEGG (Table S1). Among transcriptional regulators, SpoIIID showed the highest upregulation (3.93-fold) (Table 1). This result suggests that SpoIIID is likely a key factor in mediating the effect of *mclX* on mother cell lysis.

To verify whether SpoIIID is a key mediator of *mclX*-induced mother cell lysis, we examined SpoIIID expression levels. Western blot analysis showed that SpoIIID was detected at T8 but almost disappeared in HD73 at later stages (T12–T20). In contrast, the Δ*mclX* mutant exhibited persistently high SpoIIID levels throughout all time points examined (Fig. 1C). These results indicate that *mclX* deletion leads to sustained levels of SpoIIID, consistent with the proteomic data.

### SpoIIID modulates the process of mother cell lysis

Previous findings showed that deletion of *mclX* blocks mother cell lysis (23) and markedly upregulates SpoIIID (Fig. 1C). To investigate the impact of *spoIIID* overexpression on mother cell lysis, *spoIIID*-overexpressing strains were constructed. Three promoters with distinct temporal expression patterns were used to drive SpoIIID. P*spoIIID* is the native promoter, reflecting physiological expression. P*exsB* and P*exsY* are σ^K^-dependent promoters active during late sporulation (33, 34). P*exsB* drives expression at the late stage, while P*exsY* maintains high activity throughout the late stages (33, 34). This set of promoters enabled modulation of SpoIIID levels across different developmental stages. SpoIIID was expressed from plasmid pHT1618-based constructs under the control of different promoters.

**Fig 2:**
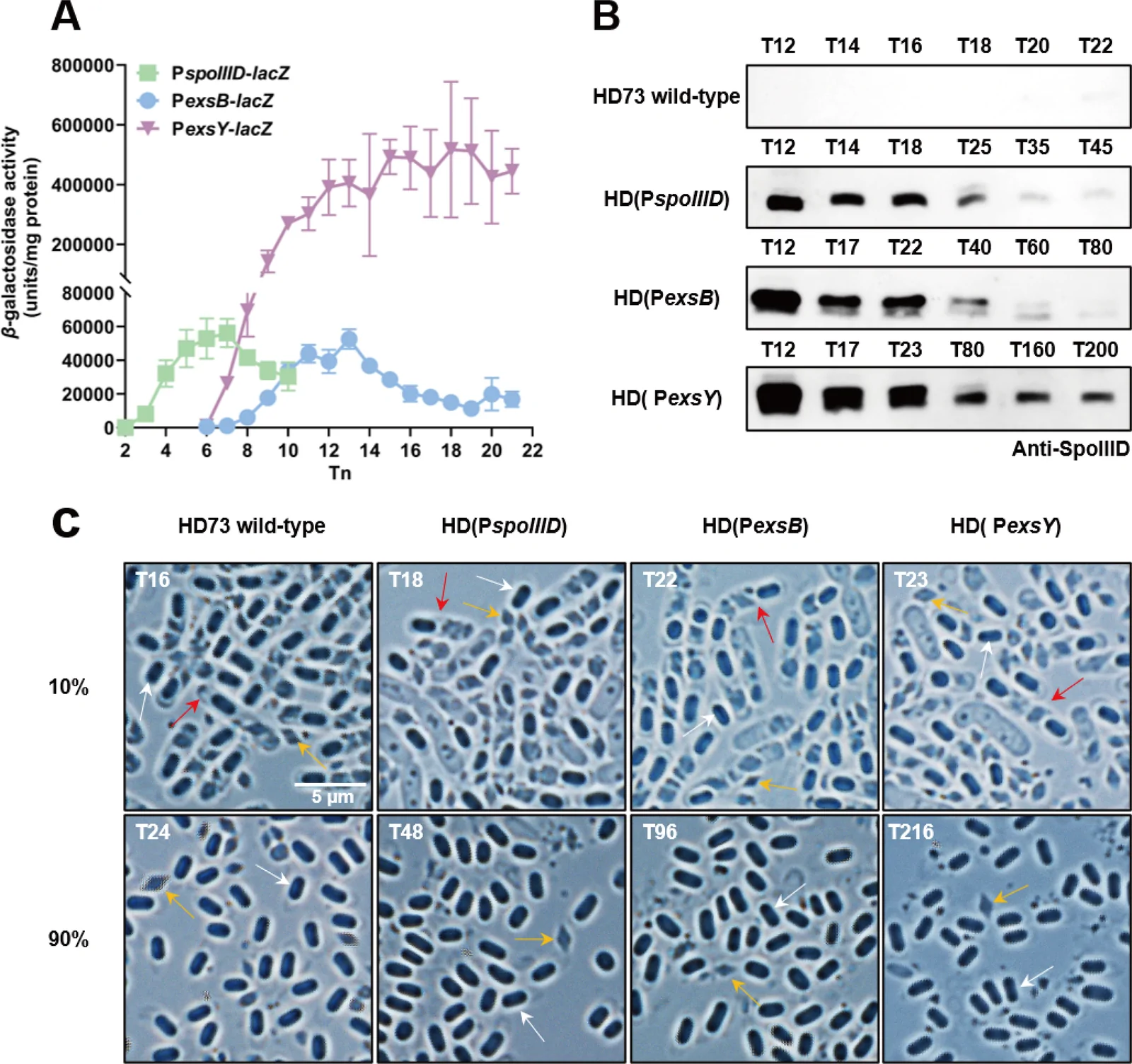
SpoIIID regulates the process of mother cell lysis. (**A**) Transcription analysis of the *spoIIID* promoter (P*spoIIID*), the *exsB* promoter (P*exsB*), and the *exsY* promoter (P*exsY*) in HD73. The β-galactosidase activities of three clones were determined at specified time points after cultivating *B. thuringiensis* cells in SSM at 30°C. Each value represents the mean and standard error of at least three independent replicates. (**B**) Western blot analysis of SpoIIID expression dynamics in HD73 and HD(P*spoIIID*), HD(P*exsB*), and HD(P*exsY*) strains. All strains were grown at 30°C in SSM with shaking. Cells were harvested at certain time points. SpoIIID antibody (Anti-SpoIIID) was used to detect the expression level of SpoIIID. (**C**) Micrographs of the HD73, HD(P*spoIIID*), HD(P*exsB*), and HD(P*exsY*) strains. All strains were grown at 30°C in SSM medium and imaged using an Olympus BX63 microscope in bright-field mode. Three structures are indicated: spores (white arrows), parasporal crystals (yellow arrows), and intact mother cells containing spores and crystals (red arrows). Lysed cells are identified by the release and dispersion of spores and crystals into the surrounding field, whereas in non-lysed cells, these structures remain enclosed within the mother cell. Scale bar, 5 μm. Mother cell lysis was assessed microscopically, with lysis defined by the release of spores and parasporal crystals from the mother cell. Lysis timing was evaluated by monitoring both the onset and completion of lysis: 10% lysis was defined as the initiation of mother cell lysis, whereas 90% lysis was defined as near-complete lysis.

To compare their differences, each promoter was fused to the *lacZ* reporter gene, and their transcriptional activities were measured in HD73. The β-galactosidase assay showed P*spoIIID* activity began at T3 (approximately 6,045 U), rose to a peak at T7 (approximately 56,178 U), and then declined. P*exsB* initiated at T8 (approximately 8,176 U) and peaked at T13 (approximately 52,613 U). By contrast, P*exsY* started at T7 (approximately 26,230 U), increased markedly to a much higher peak at T18 (approximately 516,909 U), and maintained high activity thereafter. Notably, P*exsY* activity consistently exceeded that of the other two promoters from T8 onwards, with a peak level approximately 10-fold higher (Fig. 2A). These findings reveal a stage-dependent shift in promoter activity. P*spoIIID* predominates during early sporulation, while P*exsY* drives the strongest and most sustained transcription in the late stage, and P*exsB* shows intermediate activity.

To confirm whether these three promoters effectively increase SpoIIID protein levels, we assessed SpoIIID levels using western blot. These analyses showed that SpoIIID was no longer detectable after T12 in HD73. In contrast, all three overexpression strains sustained high SpoIIID levels throughout the tested stages (Fig. 2B). These results clearly indicate that P*spoIIID*, P*exsB*, and P*exsY* successfully drove *spoIIID* overexpression.

To examine the effect of *spoIIID* overexpression on mother cell lysis, the wild-type strain HD73 was used as the control. The overexpression strains included HD73(P*spoIIID-spoIIID*), HD73(P*exsB-spoIIID*), and HD73(P*exsY-spoIIID*). Both early and extended time points were analyzed to assess changes in the overall timing of lysis. Mother cell lysis initiation was defined as the point when 10% of mother cells were visibly lysed, and complete lysis as 90% of mother cells lysed, as determined by bright-field microscopy. In HD73, mother cell lysis began at T16 and was completed by T24. In contrast, *spoIIID*-overexpressing strains required longer periods to reach comparable levels. HD73(P*spoIIID-spoIIID*) initiated lysis at T16 and completed it at T48. HD73(P*exsB-spoIIID*) began lysis at T22 and completed it at T96. HD73(P*exsY-spoIIID*) initiated lysis at T23 and reached completion only at T216 (Fig. 2C; Table S2). These results suggest that *spoIIID* overexpression delays both the initiation and completion of mother cell lysis.

To assess whether differences in lysis timing were influenced by variations in SpoIIID levels, we evaluated SpoIIID dynamics at both the initiation and completion of mother cell lysis. Western blot analysis revealed that all three overexpression strains exhibited a gradual decline in SpoIIID levels during both the initiation and progression of mother cell lysis (Fig. 2B). This indicates that elevated SpoIIID levels delay the initiation of mother cell lysis, supporting its involvement in this process.

### SpoIIID directly represses *cwlC* expression

**Fig 3:**
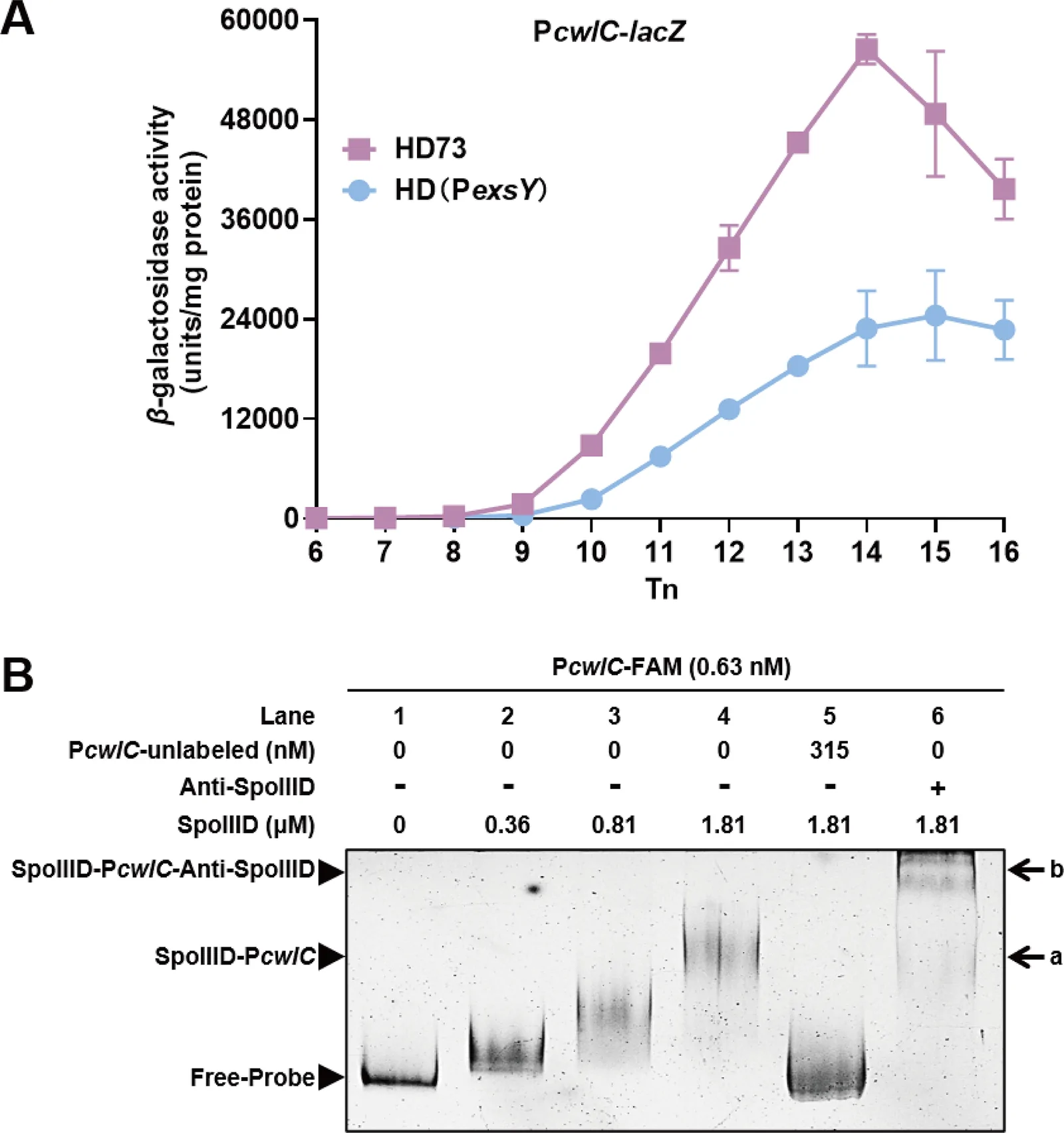
SpoIIID negatively regulates *cwlC* transcription. (**A**) Transcription analysis of the *cwlC* promoter (P*cwlC*) in wild-type HD73 and *spoIIID*-overexpression strain HD73(P*exsY*). The β-galactosidase activities of three clones were determined at specified time points after cultivating *B. thuringiensis* cells in SSM at 30°C. Each value represents the mean and standard error of at least three independent replicates. (**B**) Electrophoretic mobility shift assay (EMSA) showing binding of SpoIIID-His to the *cwlC* promoter (P*cwlC*). Lane 1, FAM-labeled P*cwlC* probe (0.63 nM) alone; lanes 2–4, probe incubated with increasing concentrations of SpoIIID; lane 5, competition assay with a 500-fold molar excess of unlabeled P*cwlC* probe; and lane 6, supershift assay with anti-SpoIIID antibody. Band a corresponds to the SpoIIID–P*cwlC*–SpoIIID antibody complex, whereas band b represents the SpoIIID–P*cwlC* complex.

Our findings showed that SpoIIID is involved in mother cell lysis, while *cwlC* is essential for this process, suggesting a regulatory link between them. To investigate this further, we examined whether sustaining the levels of SpoIIID affects *cwlC* transcription. The P*cwlC* promoter was fused to the *lacZ* reporter gene, and β-galactosidase activity was measured in both HD73 and HD73 (P*exsY–spoIIID*) strains. The results showed that *cwlC* transcription initiated at T8 and peaked at T14 in HD73. However, it was both delayed (T9) and weaker, peaking at T15 in HD73 (P*exsY–spoIIID*). Notably, P*cwlC* activity in HD73 (P*exsY–spoIIID*) remained consistently lower than that in HD73 from the onset of transcription, showing up to a 2.3-fold reduction at the peak (Fig. 3A). These results demonstrate that SpoIIID negatively regulates *cwlC* transcription.

To determine whether P*cwlC* promoter is directly or indirectly regulated by SpoIIID, SpoIIID protein was purified (Fig. S7), and EMSA experiments were performed. Shifted bands corresponding to P*cwlC*–SpoIIID complexes were detected with increasing concentrations of SpoIIID (Fig. 3B, lanes 1–4). A labeled P*cwlC* DNA probe (0.63 nM) was almost completely shifted by 1.81 μM SpoIIID, indicating that SpoIIID binds to the *cwlC* promoter DNA. A 500-fold molar excess of unlabeled P*cwlC* probe effectively competed with the labeled probe, confirming binding specificity (Fig. 3B, lane 5). Furthermore, a supershift assay using anti-SpoIIID antibodies produced a slower-migrating complex, confirming the identity of the DNA–protein complex. The upper band (band a) corresponds to the larger SpoIIID–P*cwlC*–SpoIIID antibody complex, while the lower band (band b) represents the original SpoIIID–P*cwlC* complex (Fig. 3B, lane 6). No additional distinct shifted bands were observed in the absence of antibody, indicating that SpoIIID binds specifically to the P*cwlC* promoter fragment. These results collectively indicate that SpoIIID directly represses *cwlC* transcription.

### MclX modulates mother cell lysis by SpoIIID

**Fig 4:**
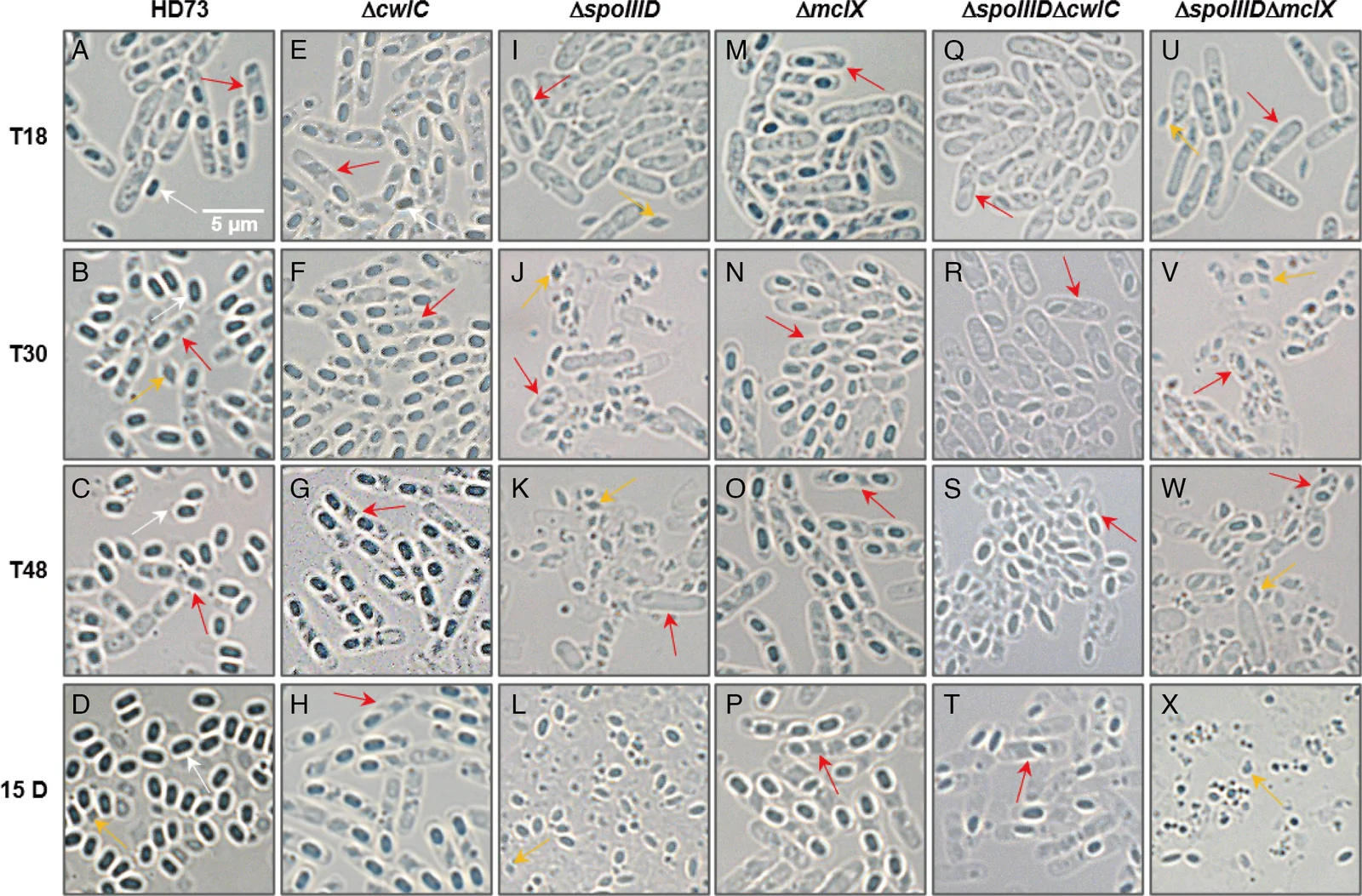
Phenotypic observation of wild-type HD73 and various *B. thuringiensis* deletion mutant strains. (**A–D**) HD73 represents the wild-type strain; (**E–H**) Δ*cwlC* represents the *cwlC* deletion mutant strain; (**I–L**) Δ*spoIIID* represents the *spoIIID* deletion mutant strain;(**M–P**) Δ*mclX* represents the *mclX* deletion mutant strain; (**Q–T**) Δ*spoIIID*Δ*cwlC* represents the *spoIIID* and *cwlC* double-deletion mutant strain; and (**U–X**) Δ*spoIIID*Δ*mclX* represents the *spoIIID* and *mclX* double-deletion mutant strain. The observation periods were T18, T30, T48, and 15 D, with T18, T30, and T48 denoting the 18th, 30th, and 48th h at the end of the logarithmic growth period of the strain, respectively (T0 is the end of the exponential growth phase, and Tn is the n hours after T0), and 15 D representing the 15th day after the transformation culture of the strain. All strains were grown at 30°C in 1/2 LB medium and imaged using an Olympus BX63 microscope in bright-field mode. Three structures are indicated: spores (white arrows), parasporal crystals (yellow arrows), and intact mother cells containing spores and crystals (red arrows). Lysed cells are identified by the release and dispersion of spores and crystals into the surrounding field, whereas in non-lysed cells, these structures remain enclosed within the mother cell. Scale bar, 5 μm.

Previous studies have shown that MclX negatively regulates SpoIIID levels (Fig. 1C), SpoIIID represses *cwlC* gene expression (Fig. 3A), and CwlC promotes mother cell lysis. To test whether MclX regulates mother cell lysis through SpoIIID, we analyzed the lysis phenotypes of relevant mutants using optical microscopy. As mother cell lysis is not detectable in the Δ*spoIIID* mutant in SSM, 1/2 Luria–Bertani (LB) medium was used to enable analysis under conditions where the phenotype can be observed (22). In the wild-type strain HD73, mother cell lysis began at T18, and the proportion of lysed cells gradually increased, reaching completion by day 15 (Fig. 4A through D). In the ∆*cwlC* mutant, no lysis was observed, even after 15 days of culture (Fig. 4E through H). The ∆*spoIIID* mutant exhibited a lysis pattern like that of the wild type, with lysis initiated at T18 and completed by day 15 (Fig. 4I through L). In contrast, the ∆*mclX* mutant showed no mother cell lysis throughout the 15-day culture (Fig. 4M through P). The ∆*spoIIID*∆*cwlC* double mutant also failed to undergo lysis (Fig. 4Q through T), whereas the ∆*mclX*∆*spoIIID* double mutant displayed normal lysis dynamics, with lysis beginning at T18 and completed by day 15 (Fig. 4U through X). These observations revealed an interesting phenomenon in the mother cell lysis process. Since deletion of *spoIIID* alone did not affect lysis, and deletion of *mclX* blocked lysis, simultaneous deletion of *mclX* and *spoIIID* restored normal lysis. Together, these results indicate that MclX functions upstream of SpoIIID and modulates mother cell lysis by SpoIIID.

### MclX regulates SpoIIID expression at a post-transcriptional level

**Fig 5:**
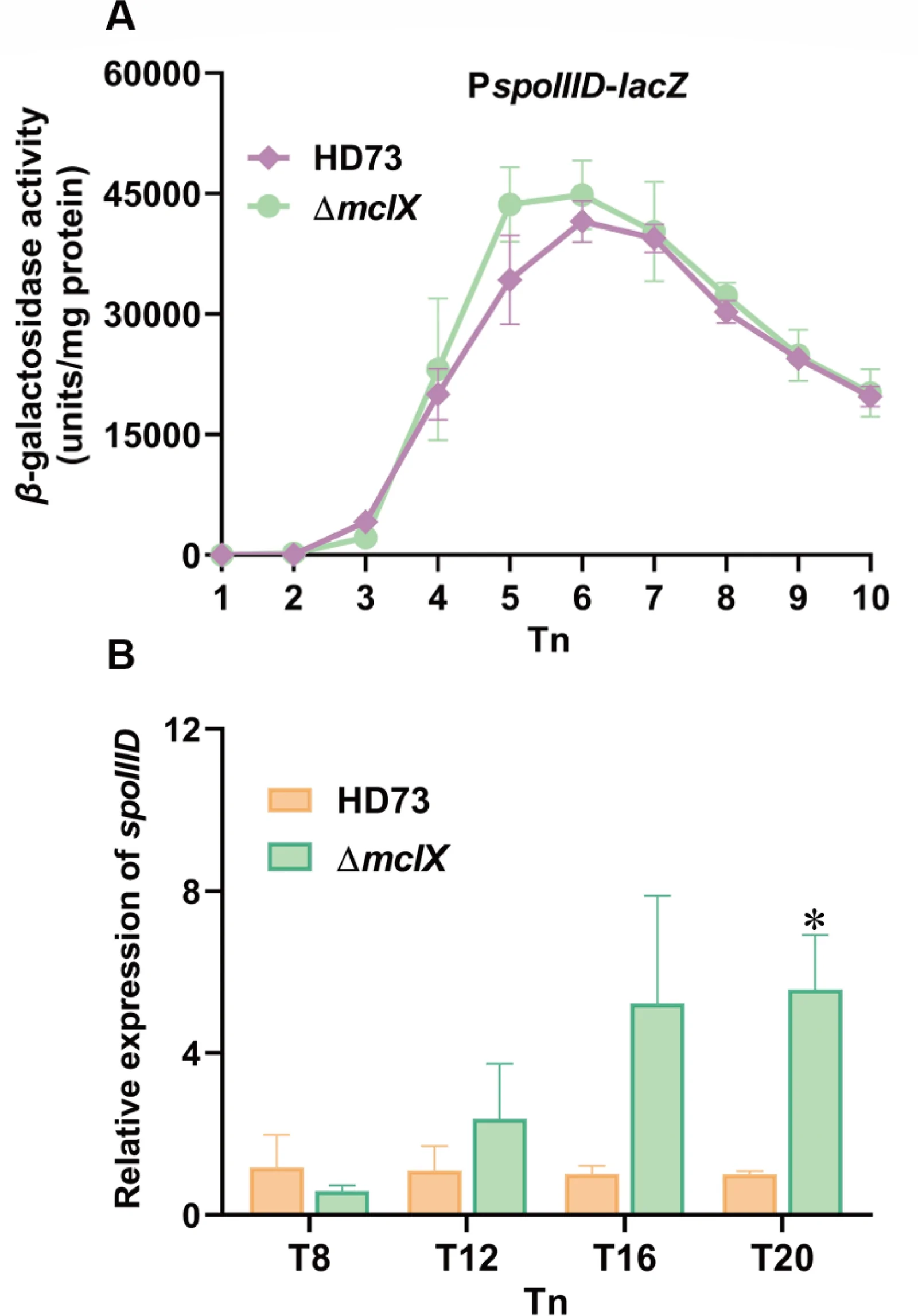
MclX regulates SpoIIID expression at a post-transcriptional level. (**A**) Transcription analysis of the *spoIIID* promoter (P*spoIIID*) in HD73 wild-type and Δ*mclX* strains. The β-galactosidase activities of three clones were determined at specified time points after cultivating *B. thuringiensis* cells in SSM at 30°C. Each value represents the mean and standard error of at least three independent replicates. (**B**) Expression profiles of the *spoIIID* gene in HD73 wild-type and Δ*mclX* strains. The cells were cultured in the SSM medium at 30°C and 220 rpm. Cells were harvested at T8, T12, T16, and T20. Error bars represent standard deviation. Single asterisks indicate significant statistical differences between two treatments at *P* < 0.05 based on Student’s *t*-test.

To investigate how MclX regulates SpoIIID expression, we examined both *spoIIID* transcriptional activity and mRNA levels. β-Galactosidase assays using a P*spoIIID-lacZ* fusion showed no significant difference between the wild-type strain HD73 and the Δ*mclX* mutant (Fig. 5A), indicating that *mclX* deletion does not affect *spoIIID* transcription. In contrast, RT-qPCR analyses revealed that *spoIIID* transcript levels were significantly increased in the Δ*mclX* mutant at T20 (Fig. 5B). This discrepancy between promoter activity and transcript abundance suggests that MclX regulates SpoIIID expression at a post-transcriptional level.

### Deletion of the *mclX* homolog in *B. subtilis* 168 does not affect mother cell lysis

**Fig 6:**
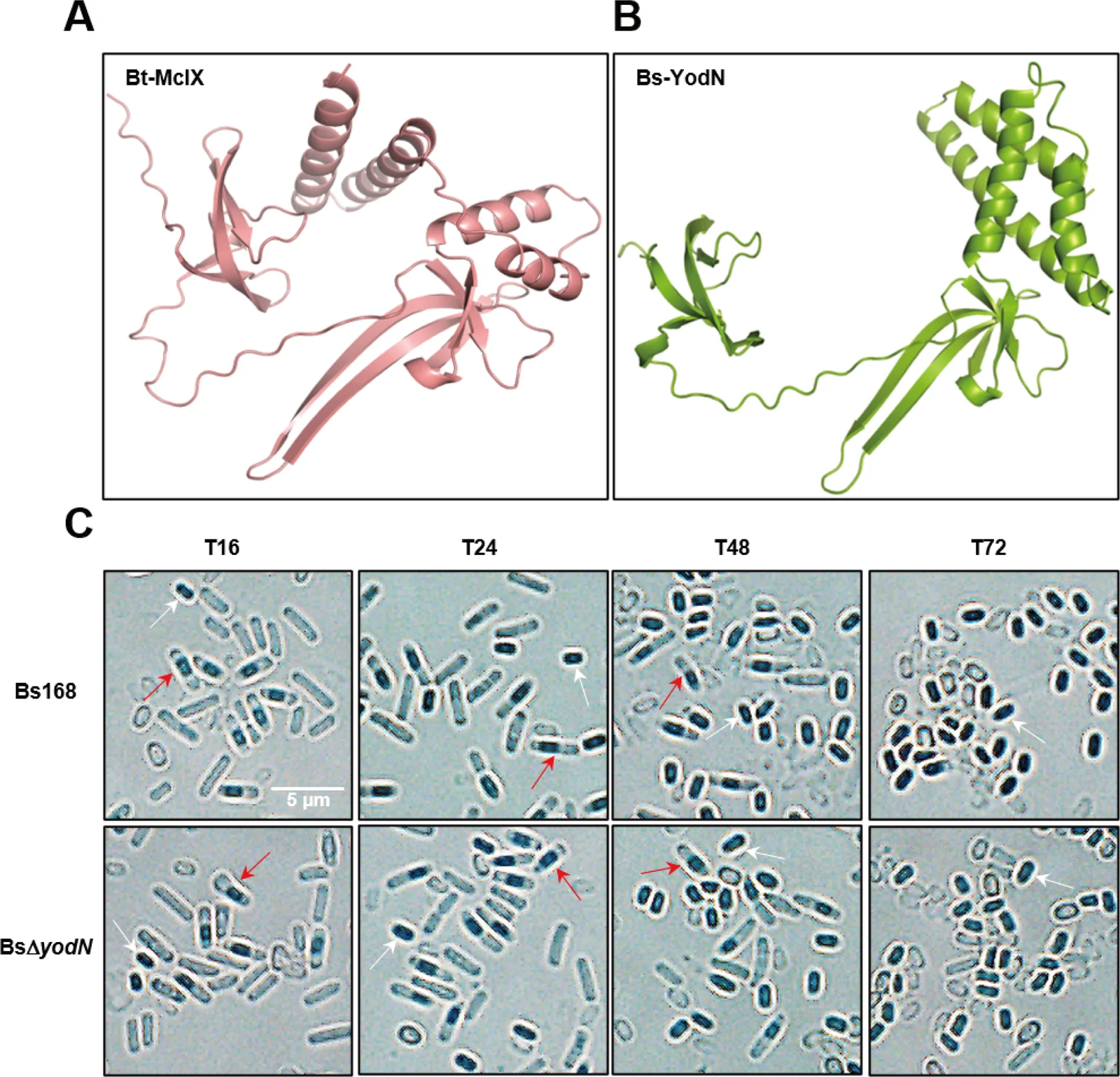
Deletion of *yodN* does not affect mother cell lysis. (**A**) Structural prediction of *B. thuringiensis* HD73-MclX. (**B**) Structural prediction of *B. subtilis* 168-YodN. (**C**) Micrographs of the *B. subtilis* 168 wild type and BsΔ*yodN*. BsΔ*yodN* represents the *yodN* deletion mutant strain. All strains were grown at 30°C in SSM medium and imaged using an Olympus BX63 microscope in bright-field mode. Two structures are indicated: spores (white arrows) and intact mother cells containing spores (red arrows). Lysed cells are identified by the release and dispersion of spores into the surrounding field, whereas in non-lysed cells, spores remain enclosed within the mother cell. Scale bar, 5 μm.

Previous studies showed that deletion of *mclX* blocked mother cell lysis in *B. thuringiensis* HD73, a member of the *B. cereus* group. To assess whether this function is conserved, we identified YodN, a structural homolog of MclX, in the model organism *B. subtilis* 168 (Fig. S5). YodN has been previously reported to affect spore germination (24). Structural prediction further revealed that HD73-MclX and *B. subtilis* 168-YodN share similar architectures, each consisting of three regions rich in β-sheets and α-helices (Fig. 6A and B). We then constructed a Δ*yodN* deletion mutant in *B. subtilis* 168 to test functional conservation. Optical microscopy revealed that both the wild-type *B. subtilis* 168 and ∆*yodN* initiated mother cell lysis at T16 and completed lysis by T72 (Fig. 6C). These results indicate that deletion of the *mclX* homolog *yodN* had no effect on mother cell lysis in *B. subtilis* 168.

## DISCUSSION

**Fig 7:**
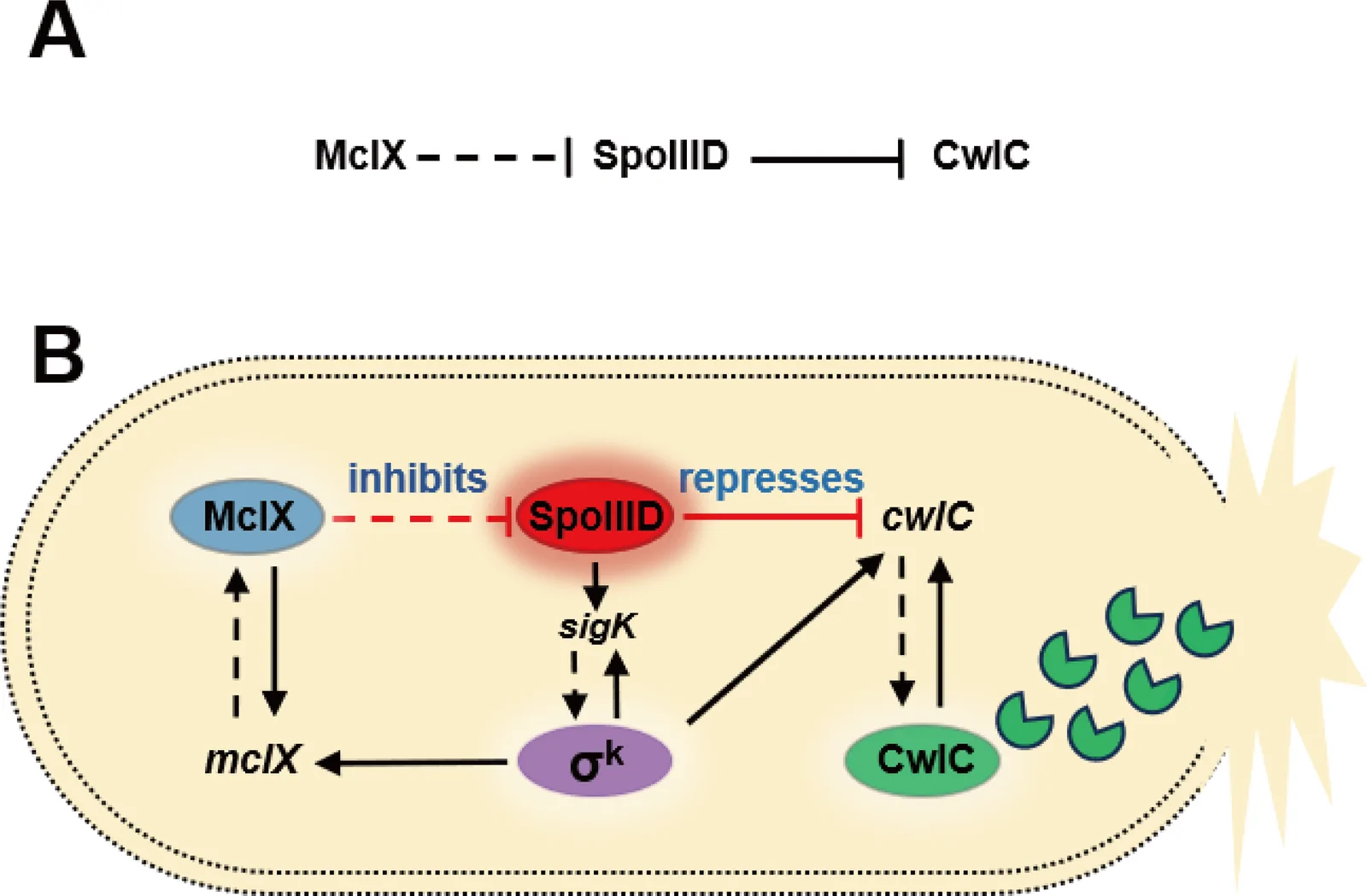
Regulatory model of MclX–SpoIIID–*cwlC* in mother cell lysis of *B. thuringiensis*. (**A**) Simplified genetic regulatory model highlighting the core pathway identified by genetic interaction analysis. MclX negatively regulates SpoIIID, which in turn represses *cwlC*, thereby controlling mother cell lysis. (**B**) Expanded regulatory network illustrating additional factors and interactions.

In this study, we revealed a previously uncharacterized regulatory pathway modulating the process of mother cell lysis in *B. thuringiensis*. Our data show that MclX negatively regulates SpoIIID, which directly represses *cwlC* transcription (Fig. 7). Previous studies have identified several critical factors involved in mother cell lysis, including the hydrolase genes *cwlB*, *cwlC*, and *cwlH* (14–19), as well as the transcriptional factor *sigK* (42). However, the mechanism modulating the process of mother cell lysis remains unclear. In *B. subtilis*, the sporulation regulatory factor σ^E^, SpoIIID, and σ^K^ control a large mother cell-specific transcriptional program, indirectly influencing the expression of mother cell lysis hydrolase genes (20). In contrast, in *Clostridioides difficile*, analogous regulators directly activate late sporulation genes’ expression, but it is not clear whether they specifically control mother cell lysis. Our findings fill this knowledge gap. MclX inhibits SpoIIID, and SpoIIID directly represses *cwlC*. Together, these interactions define an MclX–SpoIIID–*cwlC* regulatory pathway that regulates mother cell lysis.

SpoIIID performs distinct functions during the late stages of sporulation in different *Bacillus* species. In *C. difficile*, SpoIIID activates genes required for cortex and coat assembly but does not directly regulate mother cell lysis (43). In *B. subtilis*, it mainly acts as a σ^K^-dependent transcriptional regulator involved in coat formation (20). Our findings demonstrate that in *B. thuringiensis*, SpoIIID directly represses *cwlC* transcription (Fig. 3) and delays the initiation and progression of mother cell lysis when overexpressed (Fig. 2C; Table S2). Notably, no previous studies in other *Bacillus* species have reported an association between SpoIIID levels and mother cell lysis. Interestingly, in *B. subtilis*, sustained SpoIIID expression disrupts spore coat assembly and alters the expression of σᴷ-dependent genes, including *cotC* and *cotD*, leading to a reduction in spore resistance to heat and lysozyme (44). Similarly, in *B. thuringiensis*, *spoIIID* overexpression led to spore coat defects (Fig. S1), resembling the phenotype observed in *B. subtilis*. Importantly, *spoIIID* overexpression did not affect vegetative growth or sporulation efficiency (Fig. S2 and S3).

In addition to SpoIIID, proteomic analysis revealed broader effects of *mclX* deletion. All differentially expressed proteins identified in the Δ*mclX* mutant, together with their functional classification, are provided in Table S1. Given that *mclX* is a key, previously uncharacterized gene required for mother cell lysis (23), we focused on proteins associated with sporulation and mother cell lysis. Within the sporulation category, 10 proteins were differentially expressed (six upregulated and four downregulated), whereas three proteins in the mother cell lysis category were identified, all of which were downregulated (Table S1). Besides, σ^K^-associated factors were also altered in the Δ*mclX* mutant. Consistent with previous studies showing that SpoIIID positively regulates *sigK* transcription, both SpoIIID and SigK were increased in the Δ*mclX* mutant (Table S1). Notably, HD73_RS15545 and HD73_RS15875, which have been previously reported as σ^K^-regulated proteins (17, 45), were also differentially expressed in our data set (Table S1). Together, these findings indicate that the impact of *mclX* deletion extends beyond SpoIIID and likely affects the broader late sporulation regulatory network, providing candidates for future functional analysis.

Structural prediction indicates that MclX comprises three domains, consisting of Domain I with four β-sheets, Domain II with five β-sheets and two α-helices, and Domain III with two α-helices (Fig. S5A). Domains I and II match the SH3-like barrel fold within the Gene3D hierarchy. SH3 domains typically mediate protein-protein interactions and often link signaling components to membrane or cytoskeletal structures (46). Domain III is predicted to form a coiled-coil motif, which promotes protein oligomerization and confers structural stability (47, 48). These features suggest that MclX may function as a scaffolding protein. It likely oligomerizes and recruits an interaction partner through its SH3-like domains to promote the degradation or translational repression of *spoIIID* mRNA.

*B. subtilis* 168 is a well-established model strain for sporulation, and structural predictions identified YodN as the *B. subtilis* 168 homolog of Bt-MclX. YodN exhibits a similar three-domain architecture to Bt-MclX. Domains I and II each contain five β-sheets, and Domain III comprises four α-helices (Fig. S5B). The two proteins share 53% nucleotide identity and 38% amino acid identity (Fig. S6A and B). Their structural alignment yielded a TM-score of 0.4 and an RMSD of 4.83, indicating moderate structural similarity (Fig. S6C). Despite this structural similarity, a clear functional difference is apparent. Previous work showed that YodN affects spore germination in *B. subtilis* (24). In contrast, deletion of *yodN* did not affect lysis in our analysis (Fig. 6C), suggesting that MclX contributes differently to mother cell lysis in *B. thuringiensis* and *B. subtilis*. However, compensatory pathways in *B. subtilis* may mask the effect of YodN. Whether YodN influences *spoIIID* mRNA levels in *B. subtilis* remains unresolved and requires further investigation.

In summary, we propose a regulatory model in which MclX controls mother cell lysis through SpoIIID (Fig. 7). Deletion of *mclX* led to elevated SpoIIID levels, indicating that MclX negatively regulates SpoIIID levels. We further demonstrated that SpoIIID directly inhibits the expression of *cwlC*, which encodes a hydrolase critical for mother cell lysis. Our data suggest that MclX regulates SpoIIID post-transcriptionally by modulating *spoIIID* transcript levels. Together, these findings define an MclX–SpoIIID–*cwlC* regulatory pathway that modulates the process of mother cell lysis. This study provides new insight into the regulation of mother cell lysis and the understanding of bacterial cell differentiation.

## Data Availability

The mass spectrometry data generated in this study are available in ProteomeXchange (accession number PXD070258). Source data are provided with this paper.

## SUPPLEMENTAL MATERIAL

Supplemental material aem.00215-26-s0001.pdf Fig. S1 to S10; Tables S1 and S2.

ASM does not own the copyrights to Supplemental Material that may be linked to, or accessed through, an article. The authors have granted ASM a non-exclusive, world-wide license to publish the Supplemental Material files. Please contact the corresponding author directly for reuse.
