# Supplementary material for: MclX modulates the process of mother cell lysis by SpoIIID in Bacillus thuringiensis
Source: Appl Environ Microbiol. 2026 Aug 11;92(9):e00215-26. doi: 10.1128/aem.00215-26 (PMC13599503; doi:10.1128/aem.00215-26)
Supplement: Supplementary file 1 [file aem.00215-26-s0001.pdf]

Supplementary Figures and Tables

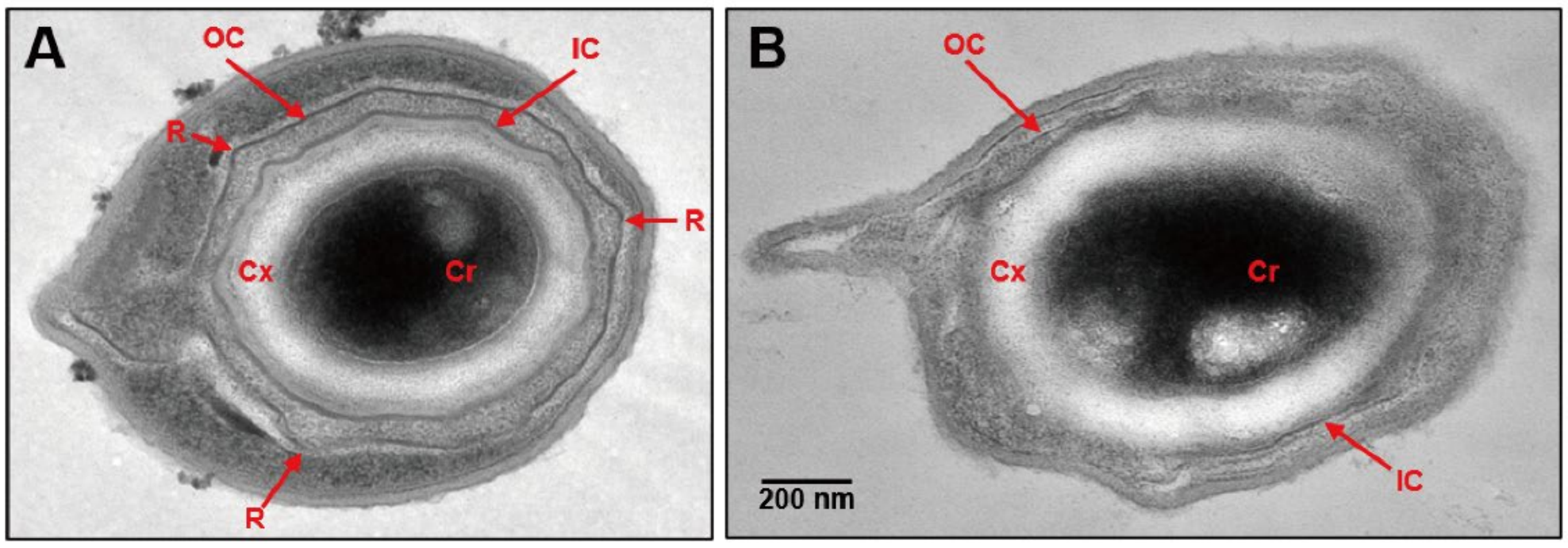

Figure S1. Transmission electron microscopic observation of Spores.

Transmission electron microscopic (TEM) observation was conducted to determine the structure of spores in HD73 wild-type (A) and SpoIIID-overexpression strain HD(*PexsY*) (B) strains at T24. The cells were cultured in the SSM medium at 30 °C and 220 rpm. The core (Cr), cortex (Cx), inner coat (IC), outer coat (OC), and ridges (R) are indicated. Bar, 200 nm.

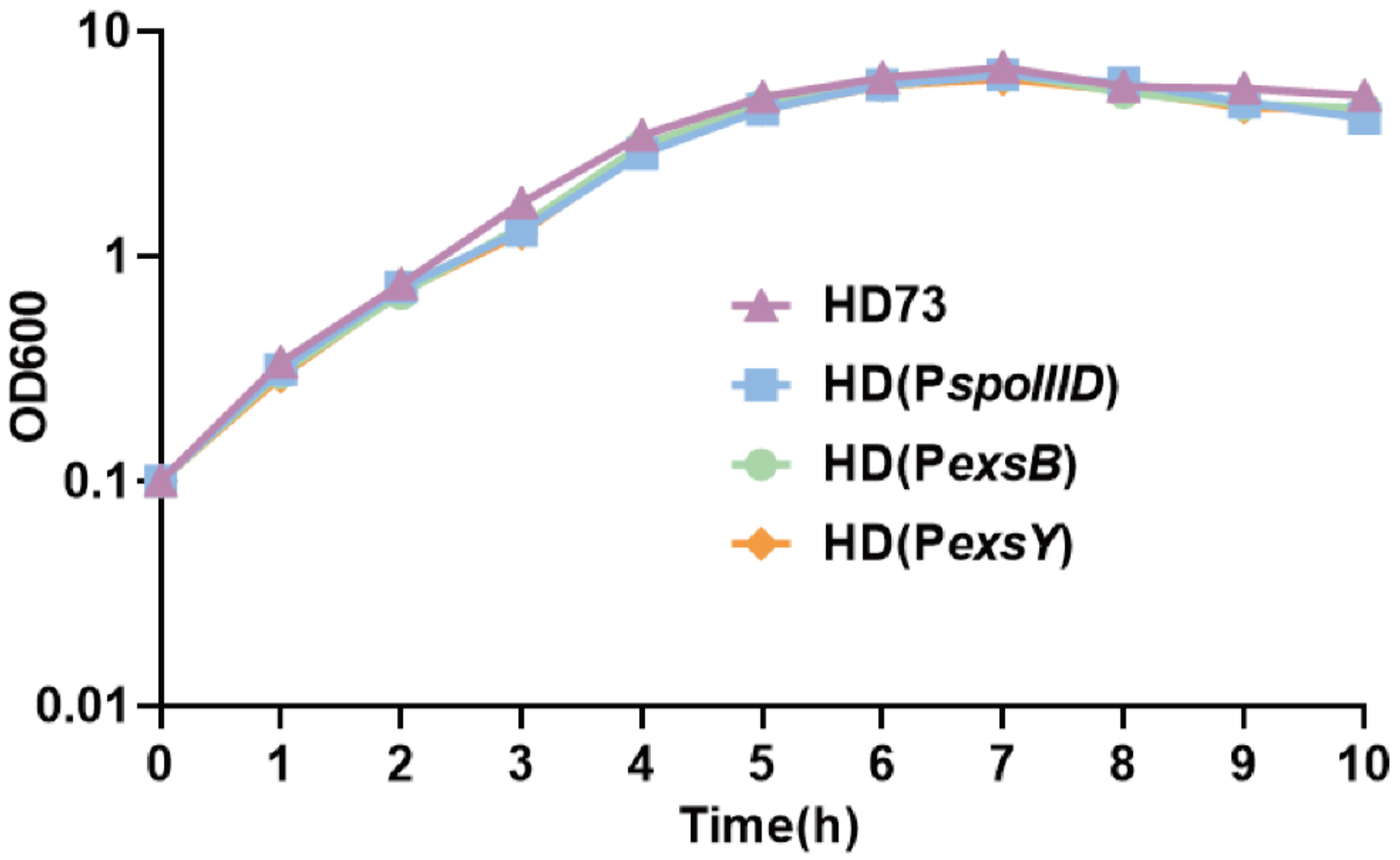

Figure S2. Growth curve of *B. thuringiensis* wild-type HD73 and SpoIIID-overexpression strains.

The cells were cultured in the SSM medium at 30 °C and 220 rpm. Next, OD600 was measured at 1 h intervals. The *y* axis represents OD600 in different strains. The *x* axis represents different time.

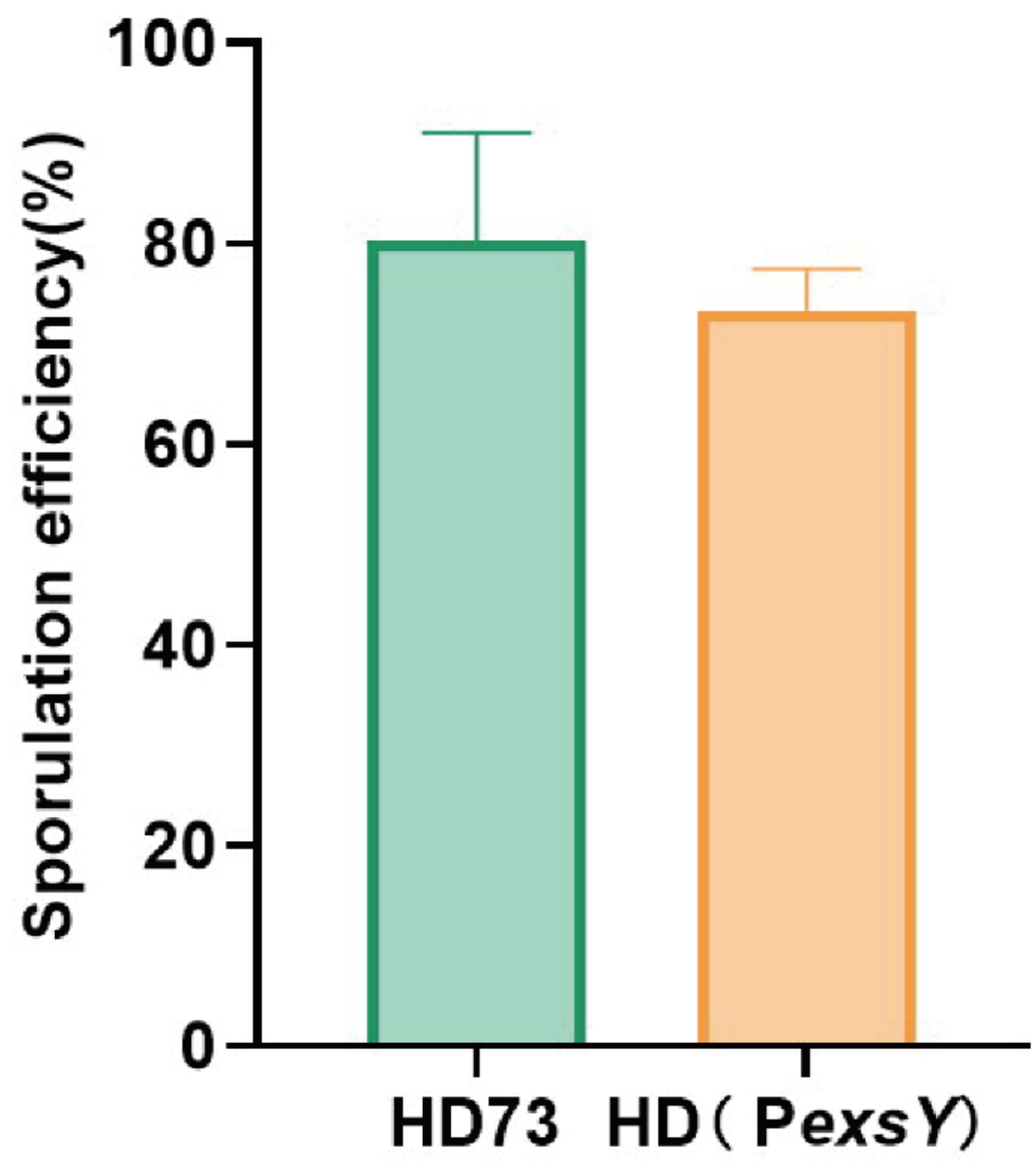

Figure S3. Effect of SpoIIID overexpression on sporulation.

Sporulation efficiencies of HD73 wild-type strain and SpoIIID-overexpression strain HD(*PexsY*) were compared. The cells were cultured in the SSM medium at 30 °C and 220 rpm. Spore counts were determined at T24. Cells were harvested and heated at 65°C for 30 min. Sporulation efficiency was defined as the ratio of the number of spores to the number of viable cells multiplied by 100. The green column represents the sporulation efficiency of the HD73 wild-type strain, and the orange column represents the sporulation efficiency of the HD(P*exsY*) strain. Data were obtained from three independent experiments, and error bars represent SD.

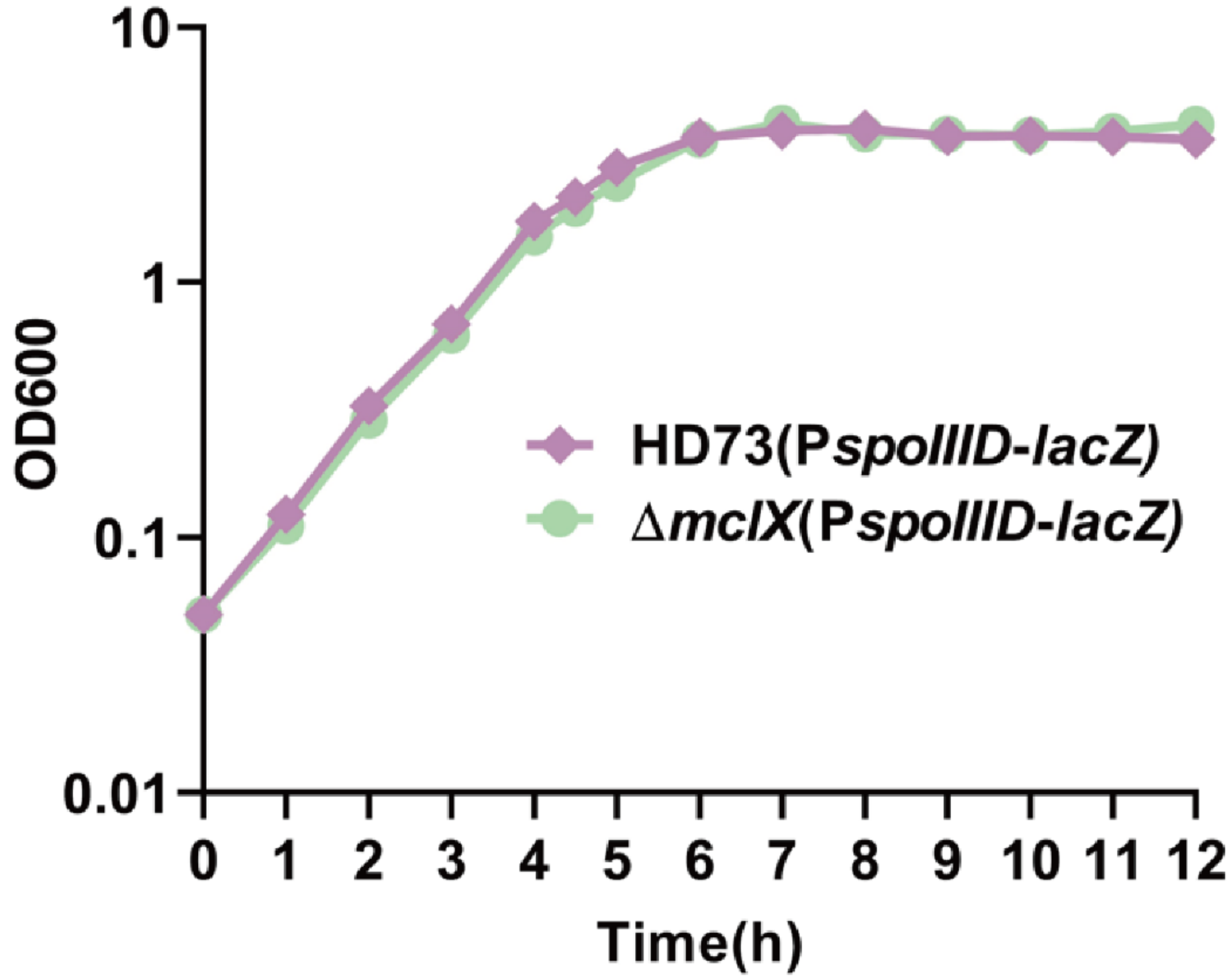

Figure S4. Growth curve of HD73(P*spoIIID-lacZ*) and Δ*mclX*(P*spoIIID-lacZ*) mutant strains.

The cells were cultured in the SSM medium at 30 °C and 220 rpm. Next, OD600 was measured at 1 h intervals. The *y* axis represents OD600 in different strains. The *x* axis represents different time.

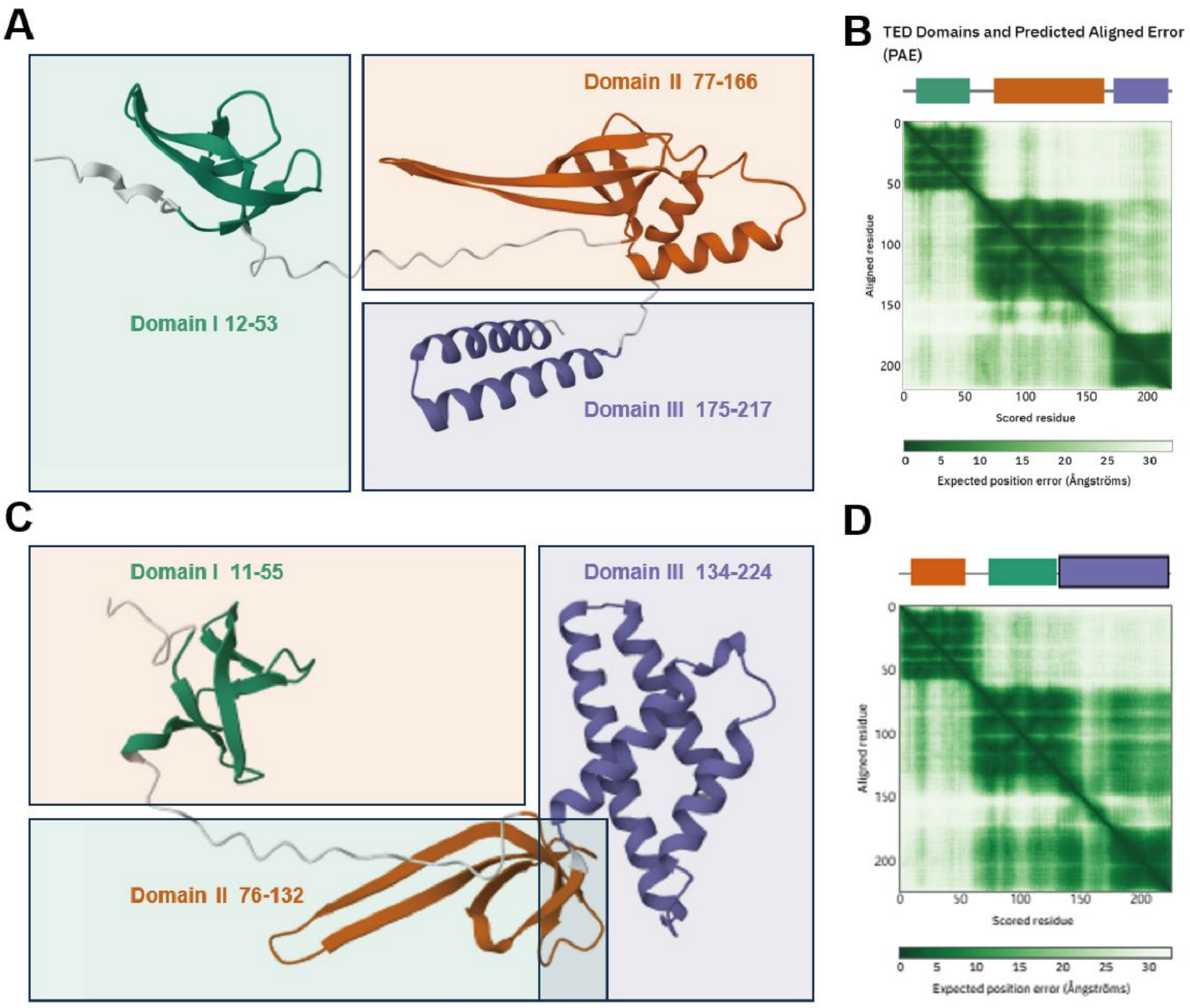

Figure S5. Predicted structure of Bt-MclX and Bs-YodN.

(A) Predicted three-dimensional structure of Bt-MclX generated by the AlphaFold Protein Structure Database. (B) Visualization of the predicted aligned error (PAE). AlphaFold shows high confidence in the relative positioning of residues 12–53 (green), 77–166 (orange), and 167–216 (purple).

(C) Predicted three-dimensional structure of Bs-YodN generated by the AlphaFold Protein Structure Database. (D) Visualization of the predicted aligned error (PAE). AlphaFold shows high confidence in the relative positioning of residues 11–55 (orange), 76–132 (green), and 134–224 (purple).

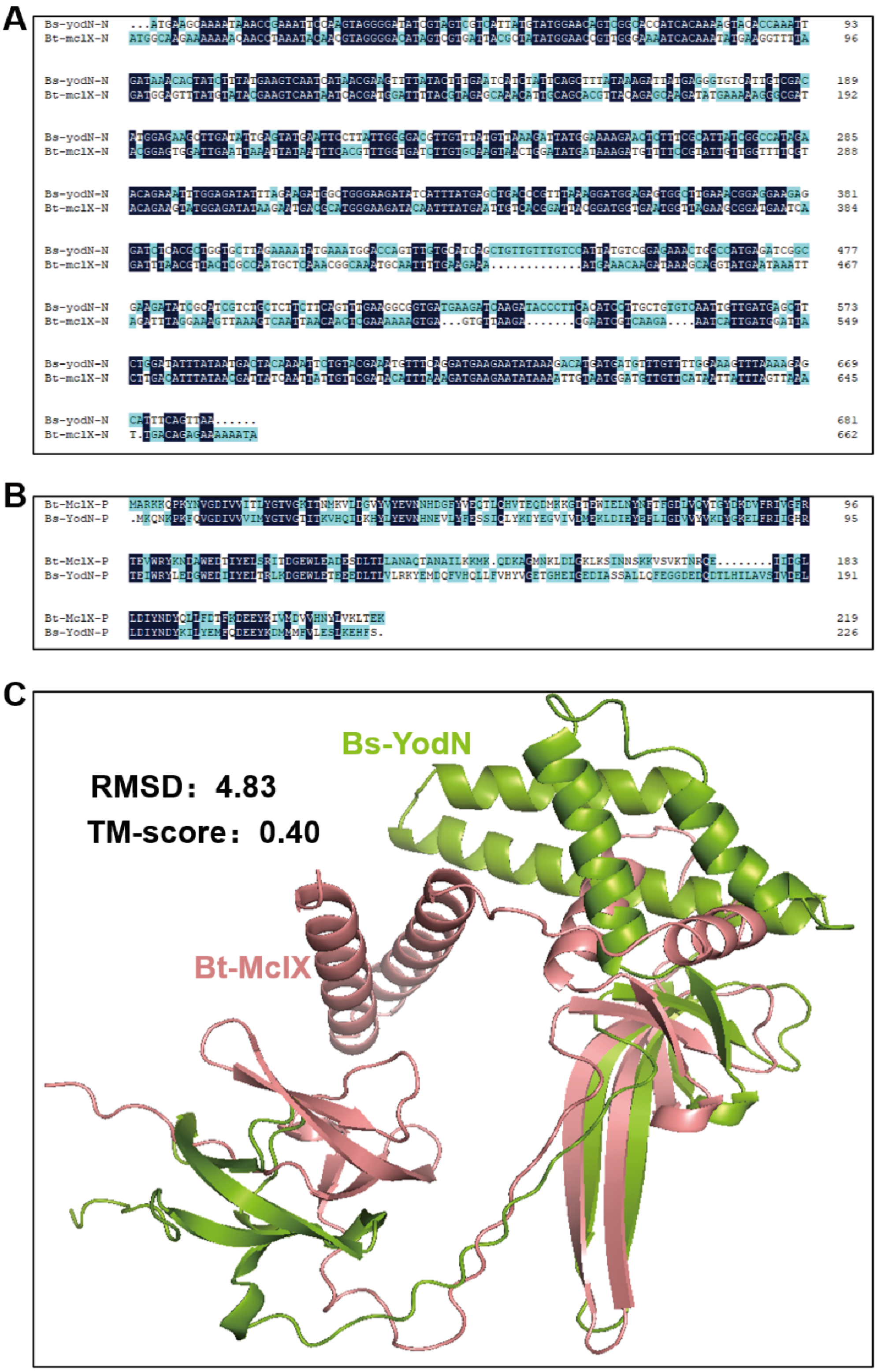

Figure S6. Sequence and Structural Alignments.

(A) Nucleotide sequence alignment. Alignment of *mclX* from *B. thuringiensis* HD73 and *yodN* from *B. subtilis* 168. The nucleotide sequence identity is 53%. (B) Amino acid sequence alignment. Alignment of MclX (*B. thuringiensis* HD73) and its homolog YodN (*B. subtilis* 168). The amino acid sequence identity is 38%. (C) Structural similarity alignment. Structural alignment between MclX and YodN. TM-score is an indicator for measuring protein structural similarity, ranging from 0 to 1. TM-score > 0.5 indicates that the two structures have similar folds, while TM-score < 0.17 suggests that the two structures are randomly similar. RMSD (Root Mean Square Deviation) measures atomic positional deviation, with lower values indicating higher structural similarity.

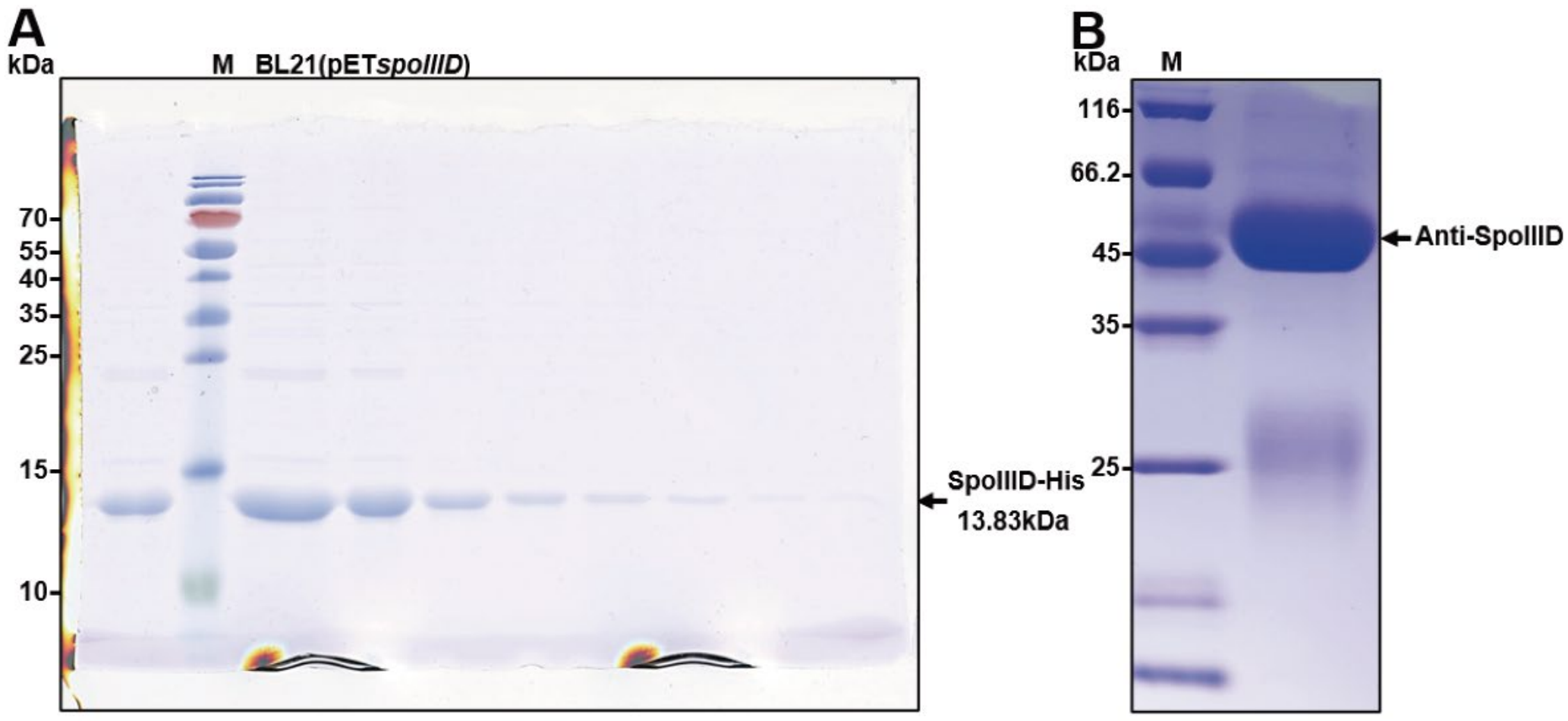

Figure S7. Purification and antibody production of SpoIIID-His recombinant protein.

(A) Original uncropped gel image for the purification of SpoIIID-His protein. (B) SDS-PAGE analysis of the purified SpoIIID antibody. The purified SpoIIID-His protein served as the antigen for antibody generation and was used as the positive control in all Western blot analyses.

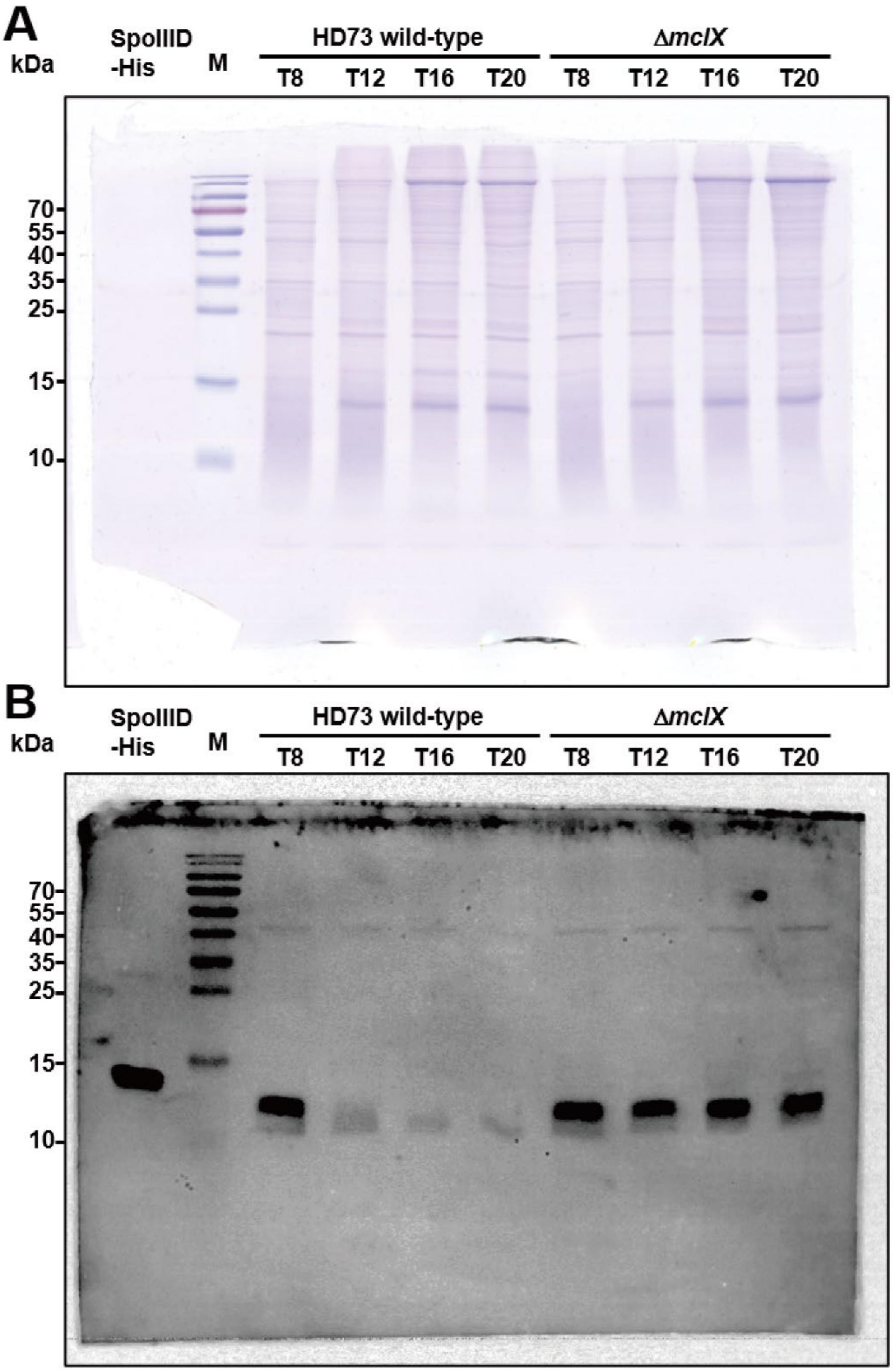

Figure S8. Original uncropped gel and Western Blot images corresponding to Figure 1C.

This file contains the full, uncropped gel and blot images used to assess SpoIIID expression levels in the HD73 wild-type and Δ*mclX* strains. (A) The full, uncropped Coomassie Brilliant Blue stained gel used for Total Protein Quantification (loading control surrogate) for samples harvested at T8, T12, T16, and T20. T0 is the end of the exponential phase, and Tn is n hours after T0. (B) The full, uncropped Western Blot image showing the detection of SpoIIID protein levels in the corresponding samples. Purified SpoIIID-His was included as a positive control.

Culture Conditions: Cells were cultured in SSM medium at 30 °C and 220 rpm.

Methodology Note: Since a suitable internal loading control (housekeeping protein) was unavailable, total protein load was normalized by running two identical SDS-PAGE gels: one stained with Coomassie Brilliant Blue for quantification (A) to ensure equal loading, and the other used for Western Blot analysis (B).

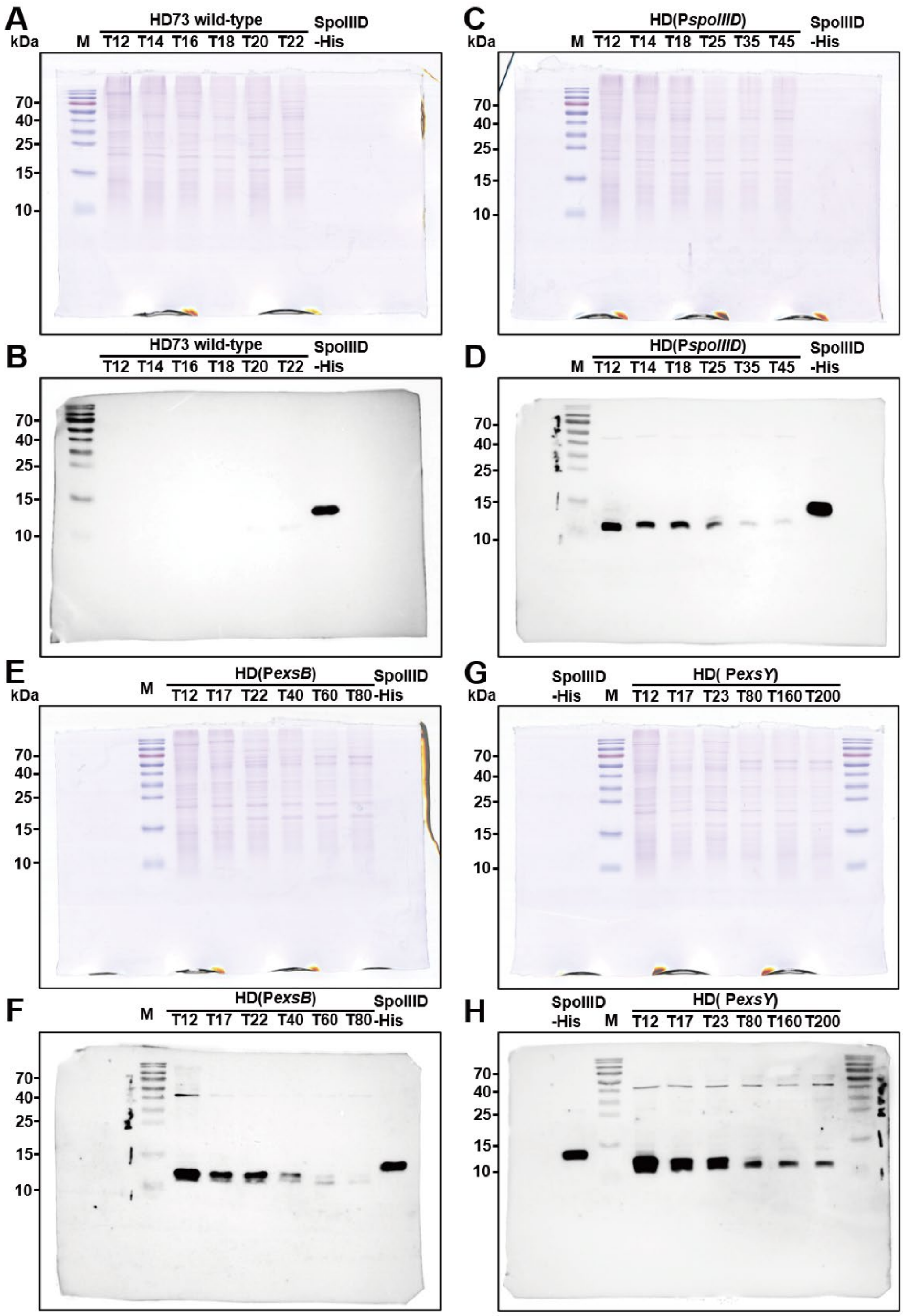

Figure S9. Original uncropped gels and Western Blot images for SpoIIID corresponding to Figure 2B.

This file contains the full, uncropped gel and blot images used to analyze the dynamics

of SpoIIID expression in various strains. (A, B) Full uncropped total-protein gel (A) and Western blot (B) showing SpoIIID levels in HD73 wild-type at T12, T14, T16, T18, T20, and T22. (C, D) Uncropped total-protein gel (C) and Western blot (D) for SpoIIID-overexpression strain HD(P*spoIIID*) at T12, T14, T18, T25, T35, and T45. (E, F) Uncropped total-protein gel (E) and Western blot (F) for HD(P*exsB*) at T12, T17, T22, T40, T60, and T80. (G, H) Uncropped total-protein gel (G) and Western blot (H) for HD(P*exsY*) at T12, T17, T23, T80, T160, and T200. Purified SpoIIID-His was used as the positive control. Total-protein staining was used as a loading control because no suitable internal reference protein was available. T0 is the end of the exponential phase, and Tn is n hours after T0. Cells were cultured in SSM medium at 30 °C and 220 rpm.

Methodology Note: Since a suitable internal loading control (housekeeping protein) was unavailable, total protein load was normalized by running two identical SDS-PAGE gels: one stained with Coomassie Brilliant Blue for quantification to ensure equal loading, and the other used for Western Blot analysis.

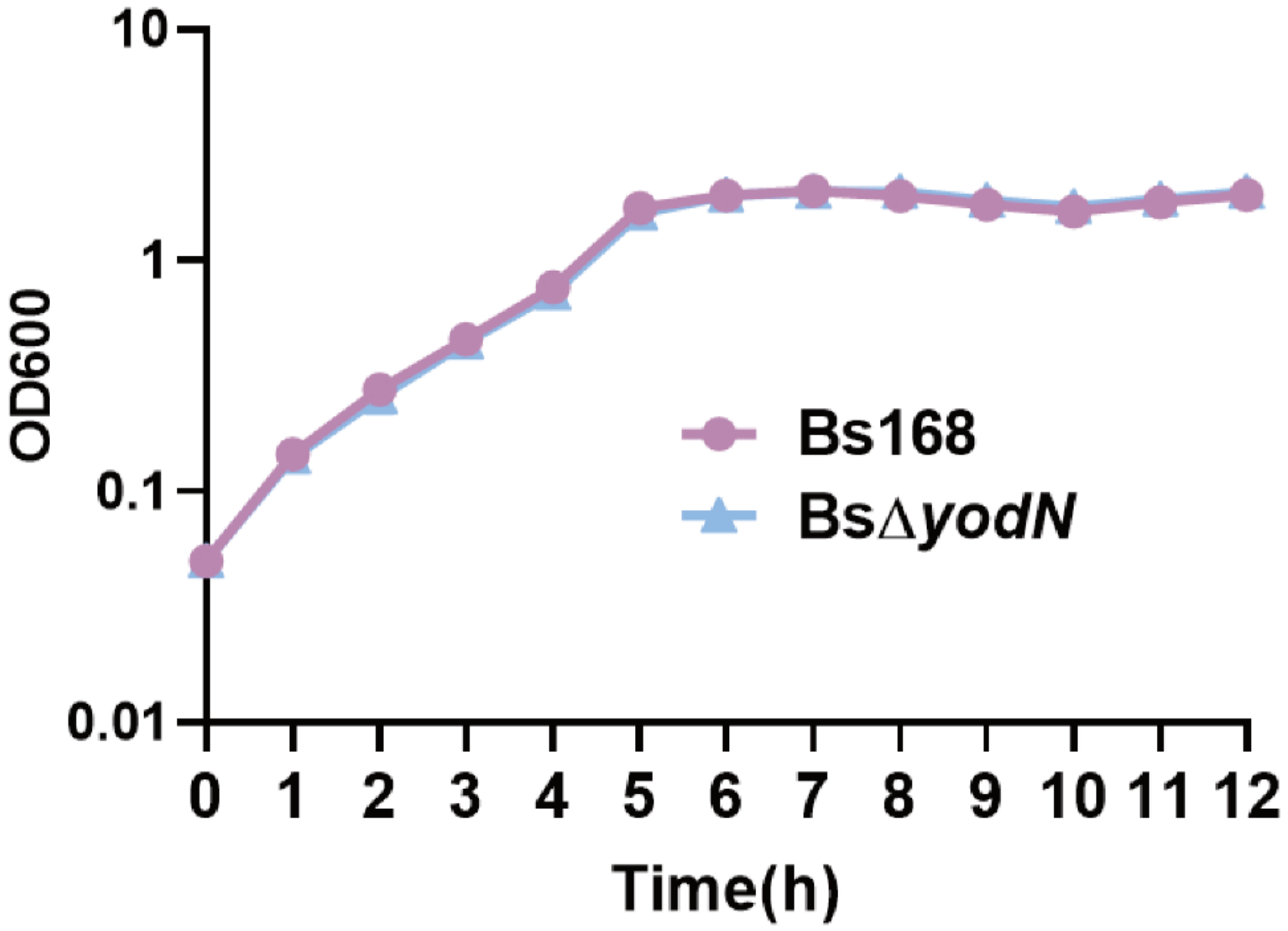

Figure S10. Growth curve of *B. subtilis* 168 wild-type and Δ*yodN* mutant strains.

The cells were cultured in the SSM medium at 30 °C and 220 rpm. Next, OD600 was measured at 1 h intervals. The *y* axis represents OD600 in different strains. The *x* axis represents different time.

Supplementary Table 1. Proteomic analysis of differentially expressed proteins between HD73 wild-type and Δ*mclX* mutant strains at T12

This table summarizes proteins showing significant differential abundance, identified by LC–MS/MS analysis. Student's T-test Difference and functional annotations are provided. T0 is the end of the exponential growth phase, and T12 is the 12 hours after T0.

**Carbohydrate metabolism**	**Upregulated genes**	
Genes ID	Annotations	Student's T-test Difference
HD73_RS28600	class II fructose-bisphosphate aldolase	2.56
HD73_RS27990	PTS lactose/cellobiose transporter subunit IIA	2.23
HD73_RS25960	glucose-6-phosphate isomerase	2.14
HD73_RS19050	aldehyde dehydrogenase DhaS	1.99
HD73_RS20485	ADP-forming succinate-CoA ligase subunit beta	1.96
HD73_RS12750	alcohol dehydrogenase AdhP	1.93
HD73_RS14460	zinc-dependent alcohol dehydrogenase	1.63
HD73_RS19770	transketolase	1.63
HD73_RS18110	phosphogluconate dehydrogenase (NAD(+)-dependent, decarboxylating)	1.56
HD73_RS18090	lactonase family protein	1.54
HD73_RS24380	6-phosphofructokinase	1.52
HD73_RS18115	transketolase	1.50
HD73_RS22340	acetyl-CoA carboxylase biotin carboxylase subunit	1.47
HD73_RS10705	L-lactate dehydrogenase	1.45
HD73_RS15180	malate:quinone oxidoreductase	1.28
HD73_RS28505	serine hydroxymethyltransferase	1.27
HD73_RS25780	glycogen phosphorylase	1.27

HD73_RS27305	2,3-bisphosphoglycerate-independent phosphoglycerate mutase	1.23
HD73_RS25265	phosphoenolpyruvate carboxykinase (ATP)	1.21
HD73_RS07515	sugar phosphate nucleotidyltransferase	1.12
HD73_RS13085	2-methylcitrate dehydratase	1.04
HD73_RS24610	acyl-CoA synthetase MbcS	1.03
**Carbohydrate metabolism**	**Downregulated genes**	
	**Downregulated genes**	
HD73_RS08465	2-isopropylmalate synthase	-1.13
HD73_RS14640	penicillin-binding protein 2	-1.23
HD73_RS12030	VOC family protein	-2.44
HD73_RS17340	acetyl-CoA carboxylase biotin carboxylase subunit	-2.57
**Amino acid metabolism**	**Upregulated genes**	
HD73_RS05350	amino acid ABC transporter substrate-binding protein	3.81
HD73_RS26090	cytosol aminopeptidase	3.54
HD73_RS20305	aspartate kinase	2.35
HD73_RS01270	D-alanine-D-alanine ligase	2.15
HD73_RS19025	argininosuccinate lyase	1.92
HD73_RS23225	aromatic amino acid hydroxylase	1.70
HD73_RS28975	homoserine dehydrogenase	1.67
HD73_RS09210	aspartate transaminase AspB	1.62
HD73_RS15820	4-hydroxy-tetrahydrodipicolinate synthase	1.55
HD73_RS19605	urocanate hydratase	1.53
HD73_RS27700	thioredoxin-disulfide reductase	1.36
HD73_RS20885	UDP-N-acetylmuramoyl-L-alanyl-D-glutamate-2,6-diaminopimelate ligase	1.20
HD73_RS03800	glycine C-acetyltransferase	1.15
**Amino acid metabolism**	**Downregulated genes**	

HD73_RS15245	bifunctional 3-deoxy-7-phosphoheptulonate synthase/chorismate mutase	-1.04
HD73_RS03895	glutamine ABC transporter substrate-binding protein GlnH	-1.07
HD73_RS10415	ketol-acid reductoisomerase	-1.64
HD73_RS01255	homogentisate 1,2-dioxygenase	-1.70
**Energy metabolism**	**Upregulated genes**	
HD73_RS15860	manganese-dependent inorganic pyrophosphatase	2.01
HD73_RS22805	superoxide dismutase [Mn]	1.61
HD73_RS08295	nitronate monooxygenase family protein	1.26
**Energy metabolism**	**Downregulated genes**	
HD73_RS26480	NAD(P)/FAD-dependent oxidoreductase	-1.24
HD73_RS08915	NAD-specific glutamate dehydrogenase	-1.27
HD73_RS28465	F0F1 ATP synthase subunit delta	-1.43
HD73_RS09090	menaquinol-cytochrome c reductase cytochrome b/c subunit	-1.89
HD73_RS28460	F0F1 ATP synthase subunit alpha	-2.19
HD73_RS26110	NAD(P)/FAD-dependent oxidoreductase	-2.28
HD73_RS04500	cytochrome aa3 quinol oxidase subunit II	-3.01
HD73_RS22885	cytochrome c550	-3.11
HD73_RS21035	cytochrome c oxidase subunit II	-3.90
**Lipid metabolism**	**Upregulated genes**	
HD73_RS23985	enoyl-CoA hydratase	2.85
HD73_RS20570	3-oxoacyl-[acyl-carrier-protein] reductase	2.53
HD73_RS06300	glycerol kinase GlpK	1.82
**Nucleotide metabolism**	**Upregulated genes**	
HD73_RS00365	hypoxanthine phosphoribosyltransferase	2.69
HD73_RS28615	CTP synthase	1.63

HD73_RS00065	IMP dehydrogenase	1.38
**Nucleotide metabolism**	**Downregulated genes**	
HD73_RS01950	adenylosuccinate lyase	-1.02
HD73_RS15545	nucleoside hydrolase	-1.12
HD73_RS21900	bifunctional UDP-sugar hydrolase/5'-nucleotidase	-1.77
**Metabolism of terpenoids and polyketides**	**Upregulated genes**	
HD73_RS22825	flavodoxin-dependent (E)-4-hydroxy-3-methylbut-2-enyl-diphosphate synthase	2.34
**Xenobiotics biodegradation and metabolism**	**Upregulated genes**	
HD73_RS18935	VOC family protein	1.77
**Genetic Information Processing**	**Upregulated genes**	
HD73_RS20435	translation elongation factor Ts	3.15
HD73_RS20360	30S ribosome-binding factor RbfA	2.63
HD73_RS01280	DEAD/DEAH box helicase	2.28
HD73_RS00495	Glutamate-tRNA ligase	1.85
HD73_RS00620	50S ribosomal protein L23	1.72
HD73_RS23720	Valine-tRNA ligase	1.66
HD73_RS20795	septum site-determining protein DivIVA	1.56
HD73_RS06360	HTH-type transcriptional regulator Hpr	1.53
HD73_RS02105	Asp-tRNA(Asn)/Glu-tRNA(Gln) amidotransferase subunit GatB	1.48
HD73_RS00745	DNA-directed RNA polymerase subunit alpha	1.36
HD73_RS23335	transcription elongation factor GreA	1.34
HD73_RS17840	Histidine-tRNA ligase	1.31
HD73_RS23380	alanine-tRNA ligase	1.18
HD73_RS28570	peptide chain release factor 1	1.09
HD73_RS13210	HU family DNA-binding protein	1.06
HD73_RS29365	redox-regulated ATPase YchF	1.05

**Genetic Information Processing**	**Downregulated genes**	
HD73_RS00650	50S ribosomal protein L29	-1.03
HD73_RS00640	30S ribosomal protein S3	-1.08
HD73_RS24185	phenylalanine-tRNA ligase subunit alpha	-1.17
HD73_RS20650	methionyl-tRNA formyltransferase	-1.19
HD73_RS22980	30S ribosomal protein S21	-1.24
HD73_RS29360	30S ribosomal protein S6	-1.26
HD73_RS00625	50S ribosomal protein L2	-1.44
HD73_RS00630	30S ribosomal protein S19	-1.48
HD73_RS00605	30S ribosomal protein S10	-1.57
HD73_RS00550	50S ribosomal protein L1	-1.91
HD73_RS00025	DNA topoisomerase (ATP-hydrolyzing) subunit B	-2.15
HD73_RS33435	50S ribosomal protein L27	-2.17
HD73_RS20525	30S ribosomal protein S16	-2.39
HD73_RS00555	50S ribosomal protein L10	-2.48
HD73_RS00560	50S ribosomal protein L7/L12	-2.74
HD73_RS00685	50S ribosomal protein L6	-4.20
**Signaling and cellular processes**	**Upregulated genes**	
HD73_RS16305	ParM/StbA family protein	3.29
HD73_RS28190	Yip1 family protein	2.78
HD73_RS26720	Fe-S cluster assembly ATPase SufC	1.97
**Signaling and cellular processes**	**Downregulated genes**	
HD73_RS15360	aminoglycoside N (3)-acetyltransferase	-1.11
HD73_RS29035	carbon starvation protein CstA	-1.12
HD73_RS22005	Na+/H+ antiporter NhaC	-1.18
**Folding, sorting and degradation**	**Upregulated genes**	
HD73_RS28585	transcription termination factor Rho	1.35
HD73_RS23005	chaperone protein DnaK	1.26

**Folding, sorting and degradation**	**Downregulated genes**	
HD73_RS20290	ribonuclease J	-1.08
HD73_RS01465	chaperonin GroEL	-1.86
**Enzymes with EC numbers**	**Upregulated genes**	
HD73_RS12895	putative beta-lysine N-acetyltransferase	1.32
HD73_RS02535	alkyl hydroperoxide reductase subunit C	1.16
**RNA degradation**	**Upregulated genes**	
HD73_RS07230	S1 RNA-binding domain-containing protein	1.43
**Protein processing**	**Downregulated genes**	
HD73_RS08835	cytochrome c biogenesis protein ResB	-2.54
**Translation**	**Upregulated genes**	
HD73_RS00590	30S ribosomal protein S7	3.55
**Chaperones and folding catalysts**	**Downregulated genes**	
HD73_RS06340	peptidylprolyl isomerase PrsA	-1.86
**Degradation**	**Downregulated genes**	
HD73_RS26985	M4 family metallopeptidase	-2.86
**Mother cell lysis**	**Downregulated genes**	
HD73_RS08930	N-acetylmuramoyl-L-alanine amidase family protein	-1.21
HD73_RS28335	M23 family metallopeptidase	-2.64
HD73_RS15875	N-acetylmuramoyl-L-alanine amidase	-4.38
**Sporulation**	**Upregulated genes**	
HD73_RS22360	stage III sporulation protein AG	3.16
HD73_RS20825	YlmC/YmxH family sporulation protein	2.12
HD73_RS20320	dipicolinic acid synthetase subunit A	1.51
HD73_RS26645	YhcN/YlaJ family sporulation lipoprotein	1.41
HD73_RS28910	spore coat protein GerQ	1.38
HD73_RS24195	small acid-soluble spore protein SspI	1.15
**Sporulation**	**Downregulated genes**	

HD73_RS07355	oligopeptide ABC transporter permease	-1.01
HD73_RS05105	spore coat protein CotJC	-1.34
HD73_RS19340	acid-soluble spore protein SspN	-1.42
HD73_RS15620	spore coat protein	-3.55
**Transcriptional regulators**	**Upregulated genes**	
HD73_RS28325	sporulation transcriptional regulator SpoIIID	3.93
HD73_RS23130	RNA polymerase sporulation sigma factor SigK	2.50
HD73_RS20925	RsfA family transcriptional regulator	2.13
HD73_RS22890	RNA polymerase sigma factor RpoD	1.26
**Transcriptional regulators**	**Downregulated genes**	
HD73_RS03255	peroxide-responsive transcriptional repressor PerR	-1.12
HD73_RS29605	Transcriptional regulator, MerR; XRE-family transcriptional regulator	-1.55
HD73_RS28880	RsfA family transcriptional regulator	-2.65
**Peptidases and inhibitors**	**upregulated genes**	
HD73_RS28730	type I methionyl aminopeptidase	1.50
HD73_RS23040	GPR endopeptidase	1.38
HD73_RS22130	tripeptidase T	1.31
**Peptidases and inhibitors**	**Downregulated genes**	
HD73_RS20025	peptidase T	-1.20
HD73_RS15045	immune inhibitor A domain-containing protein	-2.29
HD73_RS07865	M6 family metalloprotease immune inhibitor InhA1	-2.55
**Transport system**	**Upregulated genes**	
HD73_RS01160	ABC transporter substrate-binding protein	1.65
HD73_RS19210	peptide ABC transporter substrate-binding protein	1.34
HD73_RS03775	ABC transporter substrate-binding protein	1.01
**Transport system**	**Downregulated genes**	

HD73_RS17890	ABC transporter ATP-binding protein	-1.85
HD73_RS25350	ABC transporter substrate-binding protein	-2.29
**Others and unknown function**	**Upregulated genes**	
HD73_RS21005	YugN family protein	3.94
HD73_RS24995	DUF3973 domain-containing protein	3.78
HD73_RS07260	DUF3813 domain-containing protein	3.27
HD73_RS07480	class I SAM-dependent methyltransferase	3.01
HD73_RS12785	glycosyltransferase	2.74
HD73_RS27235	hypothetical protein	2.19
HD73_RS00985	YusW family protein	2.08
HD73_RS22975	GatB/YqeY domain-containing protein	1.99
HD73_RS10395	SDR family oxidoreductase	1.89
HD73_RS13095	acyl-CoA dehydrogenase family protein	1.86
HD73_RS13565	toxic anion resistance protein	1.83
HD73_RS16850	macrolide family glycosyltransferase	1.74
HD73_RS20285	translocation-enhancing protein TepA	1.68
HD73_RS04700	CBS domain-containing protein	1.59
HD73_RS08430	YkuS family protein	1.54
HD73_RS07500	bifunctional 2-polyprenyl-6-hydroxyphenol methylase/3-demethylubiquinol 3-O-methyltransferase UbiG	1.53
HD73_RS17730	alpha/beta fold hydrolase	1.41
HD73_RS09275	cell division regulator GpsB	1.41
HD73_RS19390	hypothetical protein	1.21
HD73_RS10800	glycosyltransferase	1.21
HD73_RS17320	acyl-CoA carboxylase subunit beta	1.19
HD73_RS02005	SPFH domain-containing protein	1.18
HD73_RS17675	toxin-antitoxin system YwqK family antitoxin	1.13
HD73_RS07475	glycosyltransferase	1.12
HD73_RS00955	DUF6612 family protein	1.06

HD73_RS15805	FAD-binding oxidoreductase	1.06
HD73_RS26550	NifU family protein	1.05
HD73_RS24170	YuzL family protein	1.03
HD73_RS25455	DUF4178 domain-containing protein	1.03
**Others and unknown function**	**Downregulated genes**	
HD73_RS20145	hypothetical protein	-1.07
HD73_RS18015	DUF2179 domain-containing protein	-1.08
HD73_RS12000	hypothetical protein	-1.11
HD73_RS24235	M42 family metallopeptidase	-1.32
HD73_RS21645	Cof-type HAD-IIB family hydrolase	-1.38
HD73_RS18455	YojF family protein	-1.38
HD73_RS26810	acyl-CoA dehydrogenase family protein	-1.42
HD73_RS11810	macrolide family glycosyltransferase	-1.49
HD73_RS07850	biofilm matrix protein CalY	-1.56
HD73_RS26510	DUF2628 domain-containing protein	-1.60
HD73_RS28650	(Fe-S)-binding protein	-1.92
HD73_RS30430	hypothetical protein	-2.58
HD73_RS33960	hypothetical protein	-2.76
HD73_RS25175	hypothetical protein	-3.49
HD73_RS29455	Pesticidal crystal protein cry1Ac	-11.33

Note: “Student's T-test Difference” represents the fold change in protein expression of the mutant strain △*mclX* relative to the wild-type HD73 in the proteomic analysis.

Supplementary Table 2. Quantification of mother cell lysis initiation and completion across strains

Strain	Time to lysis initiation	Lysis (%) at initiation	Time to lysis completion	Lysis (%) at completion
HD73	T16	10.7 ± 0.7	T24	98.8 ± 0.7
HD(P*spoIIID*)	T18	10.7 ± 0.7	T48	98.2 ± 1.0
HD(P*exsB*)	T22	10.5 ± 0.4	T96	99.0 ± 0.7
HD(P*exsY*)	T23	10.5 ± 0.1	T216	98.6 ± 0.5

Note: T0 is the end of the exponential phase, and Tn is n hours after T0. Lysis initiation and completion were defined as the first time points at which 10% and 90% of cells were lysed, respectively. "Lysis (%) at initiation" and "Lysis (%) at completion" indicate the measured proportions of lysed cells at these time points. Lysis was assessed by bright-field microscopy. The quantified data are presented as mean ± SD.
